# A non-canonical role for HIF-1α: redirecting DGCR8 to the RNA exosome for snoRNA degradation and translational modulation

**DOI:** 10.1093/nar/gkag070

**Published:** 2026-02-02

**Authors:** Jie-Ning Li, Ming-Yang Wang, Chiao Lo, Laising Yen, Chien-Hung Yu, Yu-Jhen Lyu, Pai-Sheng Chen

**Affiliations:** Institute of Basic Medical Sciences, College of Medicine, National Cheng Kung University, Tainan 701, Taiwan; Department of Medical Laboratory Science and Biotechnology, College of Medicine, National Cheng Kung University, Tainan 704, Taiwan; Breast Medical Center, National Cheng Kung University Hospital, Tainan 704, Taiwan; Research Center for Medical Laboratory Biotechnology, National Cheng Kung University, Tainan 704, Taiwan; Department of Surgery, National Taiwan University Hospital, Taipei 100, Taiwan; Department of Surgical Oncology, National Taiwan University Cancer Center, Taipei 100, Taiwan; Department of Surgery, National Taiwan University Hospital, Taipei 100, Taiwan; Department of Pathology and Immunology, Baylor College of Medicine, Houston 77030, TX, United States; Department of Molecular and Cellular Biology, Baylor College of Medicine, Houston 77030, TX, United States; Dan L. Duncan Cancer Center, Baylor College of Medicine, Houston 77054, TX, United States; Institute of Basic Medical Sciences, College of Medicine, National Cheng Kung University, Tainan 701, Taiwan; Department of Biochemistry and Molecular Biology, College of Medicine, National Cheng Kung University, Tainan 701, Taiwan; Department of Medical Laboratory Science and Biotechnology, College of Medicine, National Cheng Kung University, Tainan 704, Taiwan; Department of Surgery, National Taiwan University Hospital, Taipei 100, Taiwan; Institute of Basic Medical Sciences, College of Medicine, National Cheng Kung University, Tainan 701, Taiwan; Department of Medical Laboratory Science and Biotechnology, College of Medicine, National Cheng Kung University, Tainan 704, Taiwan; Breast Medical Center, National Cheng Kung University Hospital, Tainan 704, Taiwan; Research Center for Medical Laboratory Biotechnology, National Cheng Kung University, Tainan 704, Taiwan

## Abstract

The RNA exosome complex (EC) is a multi-protein complex responsible for RNA surveillance. Guided by specific adaptor factors, the EC recognizes RNA species as substrates for processing or degradation. Although its basic structure and components are documented, the regulatory mechanisms that enable this fundamental machinery to respond to biological signals remain unclear. Here, we demonstrate that hypoxia-inducible factor 1-alpha (HIF-1α) brings DGCR8 to associate with the EC in an RNA-independent manner and shifts DGCR8’s RNA-binding preference toward small nucleolar RNAs (snoRNAs). This circuit triggers the snoRNA degradation without affecting their transcription or processing, ultimately impairing ribosomal RNA (rRNA) modifications such as pseudouridylation and 2′-O-methylation, compromising rRNA processing, and reducing global translation efficiency. The HIF-1α-mediated reconfiguration of DGCR8 with EC involves the release of DGCR8 and RRP6 from the nucleoli to the nucleoplasm and is conserved across multiple species, including worms and flies. Under conditions where HIF-1α is induced, we found that the MC-to-EC switch dynamically responds to hypoxic conditions and growth factor signals. In conclusion, the discovery of MC-to-EC switch reveals a multifaceted function of HIF-1α in noncoding RNA regulation, also emphasizing its non-transcriptional impact on the DGCR8-EC-mediated snoRNA degradation pathway and its consequential effects on rRNA modifications and translation, providing new insights into RNA homeostasis regulation.

## Graphical Abstract

**Graphical Abstract:**
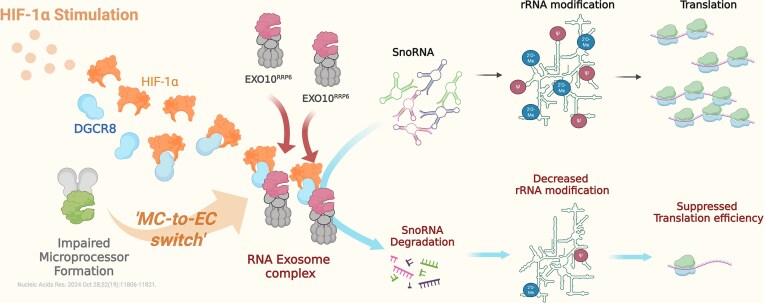

## Introduction

DGCR8 (DiGeorge syndrome chromosomal region 8) is a crucial component of the microprocessor complex (MC) involved in the biogenesis of microRNAs (miRNAs) [1]. It is a double-stranded RNA-binding protein that partners with Drosha, an RNase III enzyme, to process primary miRNAs (pri-miRNAs) into precursor miRNAs [2]. Such findings expand our view of DGCR8 as a versatile RNA-binding protein, yet the biological significance of its non-miRNA regulatory activities remains largely unexplored [3].

Hypoxia-inducible factor 1-alpha (HIF-1α) is a central transcription factor that responds to a range of stimuli, both hypoxic (low oxygen) and non-hypoxic (e.g. growth factors, reactive oxygen species, nutrient depletion) [4]. Under normoxic conditions without stimulation, HIF-1α is rapidly degraded through an oxygen-dependent mechanism: prolyl hydroxylase domain (PHD) enzymes hydroxylate specific proline residues on HIF-1α, enabling recognition by the von Hippel–Lindau E3 ubiquitin ligase complex, which targets it for proteasomal degradation [5, 6]. Through its basic-helix-loop-helix (bHLH) domain, HIF-1α transcriptionally regulates numerous genes involved in adaptation to microenvironmental stress. Other than its canonical role as a transcription factor, HIF-1α exhibits transcription-independent functions, such as promoting the interaction between CDC6 and the minichromosome maintenance complex or directly interacts with the γ-secretase complex, enhancing cancer cell metastasis and invasion [7, 8]. Recent studies have unveiled a regulatory role of HIF-1α in miRNA biogenesis: it suppresses miRNA maturation through autophagic degradation of Dicer and disrupts DGCR8 dimerization, thus impairing microprocessor assembly and repressing pri-miRNA processing [9, 10]. These additional functions underscore the complexity of HIF-1α in orchestrating gene regulation beyond its traditional role in transcription.

The eukaryotic RNA exosome is a central player in RNA biology. It is composed of nine core structural subunits (EXO9) arranged in a barrel-shaped architecture and can incorporate two main nucleases: RRP6 (EXOSC10 or PM/Scl-100 in humans) and DIS3 (RRP44) [11, 12]. Depending on the association of these nucleases, the complex may adopt distinct configurations, including EXO10^RRP6^, EXO10^DIS3^, or EXO11^RRP6+DIS3^ [13]. Through these configurations and cofactor interactions (e.g. TRAMP, PAXT, NEXT), the exosome processes a variety of RNA substrates, such as ribosomal RNAs (rRNAs), small nuclear RNAs (snRNAs), small nucleolar RNAs (snoRNAs), long noncoding RNAs (lncRNAs), and messenger RNAs (mRNAs), while selectively degrading aberrant transcripts [11]. Despite its well-established roles in fundamental RNA metabolism, the mechanisms governing the exosome’s assembly and activity in response to various biological cues remain largely unclear.

In this study, we unveil a novel mechanism by which HIF-1α drives assembly of DGCR8 with RNA exosome complex (EC), hereafter termed the “MC-to-EC switch.” We show that HIF-1α selectively enhances the interaction between DGCR8 and RRP6-containing EC. Mechanistically, this HIF-1α-mediated MC-to-EC switch induces snoRNA degradation, leading to hypo-modified rRNAs and compromised translation efficiency. Moreover, this HIF-1α-mediated MC-to-EC switch responds to pathophysiological stimulations, including hypoxia and growth factors, and is evolutionarily conserved in *Caenorhabditis elegans* and *Drosophila melanogaster*, underscoring its fundamental cross-species role in cell physiology. Collectively, our findings demonstrate the pivotal role of HIF-1α in modulating EC function post-DGCR8 engagement, filling an unknown gap in our understanding of HIF-1α’s non-transcriptional activity in noncoding RNA regulation.

## Materials and methods

### Cell culture

HEK293T cells (human embryonic kidney cell line) were maintained in Dulbecco’s Modified Eagle’s Medium/Nutrient Mixture F-12 (DMEM/F12) medium, while MDA-MB-231 cells were cultured in high-glucose Dulbecco’s modified Eagle’s medium (DMEM), and MCF-7 cells in low-glucose DMEM. All media were supplemented with 10% fetal bovine serum (Corning®) and 1% penicillin-streptomycin. Cultures were incubated at 37°C in a humidified atmosphere containing 5% CO_2_.

### Protein extraction and immunoprecipitation

Cells were rinsed twice with phosphate buffered saline (PBS) and lysed in NETN buffer [150 mM NaCl, 20 mM Tris–HCl, pH 8.0, 0.5% NP-40, and 1 mM ethylenediaminetetraacetic acid (EDTA)] supplemented with protease inhibitors. Lysates were sonicated and centrifuged at 14 000 rpm for 30 min at 4°C to remove debris. For model organisms, whole flies or worms were homogenized in NETN buffer, followed by sonication and centrifugation as above. Protein concentrations were determined by the Bradford assay. For immunoprecipitation (IP), 1 mg total protein was pre–cleared with Pierce™ Protein A Plus agarose beads for 1 h at 4°C. After bead removal (2500 rpm, 5 min), the supernatant was incubated with primary antibody overnight at 4°C. Immune complexes were captured with agarose beads for 1 h at 4 °C, washed twice with NETN buffer (2500 rpm, 5 min each), and analyzed by western blotting.

### Western blot

Protein lysates were denatured at 100°C for 10 min, separated by sodium dodecyl sulfate–polyacrylamide gel electrophoresis, and transferred to PVDF membranes (GE Healthcare). Membranes were blocked with 5% (w/v) skim milk for 1 h at room temperature (RT), incubated with primary antibodies (Supplementary Table S2) overnight at 4°C, washed three times with Tris-Buffered Saline with Tween-20 (TBST), and then incubated with Horseradish Peroxidase (HRP)–conjugated secondary antibodies for 1 h at RT. Signals were detected using an enhanced chemiluminescence (ECL) system (PerkinElmer, Waltham, MA, USA).

### Total RNA extraction

Total RNA was extracted using TRIzol (Invitrogen) according to the manufacturer’s instructions. Briefly, cells were lysed in TRIzol, mixed with chloroform, and centrifuged at 14 000 rpm for 30 min at 4°C. The aqueous phase was precipitated with isopropanol at −20°C overnight. RNA pellets were washed with 75% ethanol, air–dried, and dissolved in nuclease–free water. RNA concentration was measured using a NanoDrop Lite spectrophotometer.

### RNA dot blot

Total RNA (1 µg) was heated at 85°C for 5 min to disrupt secondary structures and immediately chilled on ice. Denatured RNA was spotted onto a positively charged nylon membrane and UV–crosslinked (254 nm, 125 mJ cm^−2^) for 15 min. Membranes were rinsed with TBST and blocked with 5% skim milk for 1 h at RT, incubated with anti–pseudouridine primary antibody (MBL, D347–3) overnight at 4 °C, washed, and probed with HRP–conjugated secondary antibodies. Signals were detected by ECL. For loading control, total RNA on the same membrane was visualized by SYBR™ Gold staining.

### Reverse transcription

Complementary DNA (cDNA) was synthesized using the M–MLV Reverse Transcriptase kit (Promega, M1701). A 12 µl mix containing 200 ng RNA, 1 µl 100 µM random hexamers, and 2 µl 10 mM deoxyribonucleoside triphosphates (dNTPs) was heated to 65°C for 5 min. Then, 4 µl 5X buffer, 0.6 µl reverse transcriptase, 0.4 µl RNasin® (Promega, N2111), and nuclease–free water were added to 20 µl total. Reactions were incubated at 25°C for 5 min, 37°C for 60 min, and 70°C for 5 min. cDNA was stored at −20°C.

### Quantitated real-time PCR

Quantitative real-time PCR (qPCR) was performed using ORA™ qPCR Green ROX H Mix (highQu, Germany) on an Applied Biosystems StepOne system. Reactions contained SYBR Green master mix, forward and reverse primers, nuclease–free water, and cDNA template. Cycling conditions were 95°C for 10 min; 40 cycles of 95 °C for 15 s and 60 °C for 1 min. For snoRNA and pri–miRNA detection, specific primers (amplicon size 50–200 bp) are listed in Supplementary Table S1. Primers for rRNA precursors and intermediates were from [14]. For actinomycin D assay, cells were treated with actinomycin D (5 µg ml^−1^) to block global transcription by inhibiting RNA polymerases I, II, and III [15–17], and were collected at the indicated time points (0, 4, and 8 h). SnoRNA stability was assessed by RT-qPCR.

### RNA immunoprecipitation

RNA immunoprecipitation (RIP) was performed following the IP procedure above. After pre–clearing, lysates were incubated with primary antibodies overnight at 4°C. Antibody–bound complexes were captured with agarose beads for 1 h at 4°C and washed twice with NETN buffer (2500 rpm, 5 min each). RNA associated with immune complexes was extracted with TRIzol, reverse–transcribed, and analyzed by qPCR [18–21].

### Reverse transcription at low deoxyribonucleoside triphosphate concentrations followed by polymerase chain reaction

RTL–P assays for detection of 2′–O–methylated rRNA sites were performed as described in [22]. Conventional RT–PCR was set up with 800 ng total RNA in 4 µl pre–RT mixture (10 µl total) containing 4 µl specific RT primer (50 µM) and 2 µl dNTPs (10 mM for high–dNTP reactions; 10 µM for low–dNTP reactions), followed by 65°C for 5 min. Next, 4 µl 5X RT buffer, 0.5 µl reverse transcriptase (Promega, M1701), 0.25 µl RNasin® (Promega, N2111), and nuclease–free water were added to 20 µl. Reactions were incubated at 42°C for 10 min, then 42°C for 60 min, and 70°C for 10 min. cDNA was amplified with PrimeSTAR® GXL DNA Polymerase (TaKaRa, R50A) using primer pairs flanking the modified sites. PCR conditions were 35 cycles of 98°C for 10 s, 60°C for 15 s, and 68°C for 1 min. Products were resolved by agarose gel electrophoresis.

### Surface sensing of translation assay

Cells of interest were incubated with puromycin (10 µg ml^−1^) for 30 min at 37°C in 5% CO_2_. Cells were then harvested for protein extraction, and translation was assessed by western blotting with anti–puromycin (MABE343, clone 12D10).

### Constructs

The HIF–1α coding sequence was cloned into pcDNA3.1, and HIF–1α truncations were generated in pcDNA3.1 by Dr Chih–Chen Hong [9]. For multi–fluorescence constructs, the cyan fluorescent protein (CFP) C–terminal fragment (CC155) was fused to the N–terminus of human DGCR8, the CFP N–terminal fragment (CN155) to RRP6, and the yellow fluorescent protein (YFP) N–terminal fragment (YN155) to Drosha in pBiFC (Biomedical Resource Core, First Core Laboratory, College of Medicine, National Taiwan University). Constructs expressing DGCR8 wild–type and point mutants (W329A and W329H) were provided by Dr Feng Guo [23]. Bimolecular fluorescence complementation (BiFC) platform plasmids (VC155–RRP6, VN173–DGCR8, VC155–HIF–1α, and VN173–RRP6) were generated by the National RNAi Core Facility at Academia Sinica (Taiwan).

### Bimolecular fluorescence complementation (BiFC) assays and multi-fluorescence complementation assay (MiFC)

For BiFC assay, two chimeric constructs were used: VN173–DGCR8, in which the N–terminal fragment of Venus (residues 1–173) was fused to the N–terminus of DGCR8; and VC155–RRP6, in which the C–terminal fragment of Venus (residues 155-end) was fused to the N–terminus of RRP6. For multi-fluorescence complementation (MiFC) assay, CC155–DGCR8, YN155–Drosha, and CN155–RRP6 were co–expressed. Cells were transfected for 24 h, lysed, and total protein lysates were collected. Fluorescence was measured on a multi–mode microplate reader equipped with appropriate filter sets (515/529 nm for Venus/YFP and 405/485 nm for CFP), following published protocols [24–27]. To avoid fluorescence resonance energy transfer between CFP and YFP, YFP signals were recorded before CFP signals. Each data point represents the population–averaged fluorescence intensity from independent biological replicates (*n* ≥ 3), with each replicate measured in duplicate.

### Transfection and shRNAs interference assay

Plasmid transfections were performed with HyFect™ DNA transfection reagent (Leadgene Biomedical, Taiwan) according to the manufacturer’s protocol. DNA and reagent were mixed in serum–free medium for 15 min and added to cells. Lentiviral particles for short hairpin RNA (shRNA) delivery were produced in HEK293T cells by co–transfecting pCMV–ΔR8.91, pMD.G, and pLKO.1–shRNA constructs. Viral supernatants were collected at 24 and 48 h, filtered, and used to infect target cells in the presence of polybrene. Protein lysates were harvested and target knockdown was confirmed by western blotting.

### Hypoxic condition, growth factor, and chemical treatment

MDA–MB–231 cells were serum–starved for 24 h and then treated with epidermal growth factor (EGF) (40 ng ml^−1^) or insulin-like growth factor (IGF) (100 ng ml^−1^) for 24 h. Hypoxia was induced by incubating cells in a 1% O_2_ chamber for 6 h. HEK293T cells were treated with cobalt chloride (CoCl_2_, 200 µM) for 24 h.

### Glycerol gradient

Glycerol gradient fractionation was performed as follows. Cells from ten 10–cm dishes at ∼80% confluence were collected in NETN lysis buffer. Linear 5%–50% (w/v) glycerol gradients were prepared in glycerol buffer (150 mM NaCl, 50 mM Tris base, 1 mM EDTA [pH 8]) with clearly defined interfaces. Cell lysates were layered on top and centrifuged for 6 h at 115 000 × *g* (Beckman, MLS–50). Twenty–six fractions were collected manually from the top.

### Nucleolar isolation

Cells from two 15–cm dishes at ∼80% confluence were washed twice with PBS and scraped into 2 ml hypotonic fractionation buffer (20 mM HEPES, 10 mM KCl, 2 mM MgCl_2_, 1 mM EDTA, 1 mM EGTA (ethylene glycol tetraacetic acid), 1 mM DTT (Dithiothreitol), protease inhibitors). Suspensions were incubated on ice for 20 min and sheared using a 3 ml syringe with a 27–G needle (number of passes depended on cell type; e.g. 5 passes for HEK293T). Nuclei were pelleted at 720 × *g* (∼3000 rpm) for 5 min; the supernatant (cytoplasm) was collected. The nuclear pellet was resuspended in 1 000 µl fractionation buffer, passed through a 3 ml syringe with a 24–G needle 10 times, and centrifuged at 720 ×* g* for 5 min. The pellet was resuspended in 500 µl S1 buffer (250 mM sucrose, 2 mM MgCl_2_), underlaid with 500 µl S2 buffer (350 mM sucrose, 0.5 mM MgCl_2_) without mixing, and centrifuged at 2500 rpm for 5 min. The pellet was resuspended in 300 µl S2 buffer and sonicated on ice (50% amplitude, six pulses) until no intact nuclei were observed microscopically. The sonicated nuclear fraction was underlaid with 200 µl 880 mM sucrose/0.5 mM MgCl_2_ and centrifuged at 3500 rpm for 10 min. The supernatant (nucleoplasm) and pellet (nucleoli) were separated. The nucleolar fraction was washed once with 500 µl S2 buffer and centrifuged at 3500 rpm for 5 min [28]. For western blotting, pellets were lysed in RIPA buffer.

### Immunofluorescence and image analysis

Cells were seeded on Millicell® EZ Slides and treated with CoCl₂ for 24 h. Cells were washed three times with PBS, fixed with 3.7% formaldehyde for 15 min, permeabilized with 0.4% Triton X–100 in PBS for 15 min, and blocked with 2% bovine serum albumin (BSA) for 1 h. Primary antibodies (1:100; anti–DGCR8, Abnova H00054487–B01P; anti–RRP6, GeneTex GTX107524) diluted in 0.1% BSA were applied overnight at 4°C. After five PBS washes, secondary antibodies (1:200) in 0.1% BSA were applied for 1 h at RT. Nuclei were stained with Hoechst (1 µg ml^−1^, 10 min). Slides were washed five times with PBS and mounted with coverslips. Images were analyzed in ImageJ. Nucleoli were manually segmented; mean intensities were measured, and nucleoplasmic mean intensity (%) was calculated as: [(integrated intensity − nucleolar intensity)/(integrated area − nucleolar area)] × 100.

### *In situ* proximity ligation assay

Interactions between DGCR8/Drosha and DGCR8/RRP6 in breast cancer tissues were assessed using tissue microarrays CBA4 (Super Bio Chips) and BRC1021 (US Biomax Inc.). Use of tissue microarrays complied with NCKU Hospital IRB approvals (IRB No. A–ER–105–491 and A–ER–106–483). Proximity ligation assay (PLA) was performed according to the manufacturer’s instructions (Sigma–Aldrich). Tissue microarrays were baked at 60°C for 1 h and rehydrated in water. Two primary antibodies from different host species were applied to the arrays and incubated overnight at 4°C (anti–DGCR8, Abnova H00054487–B01P; anti–Drosha, Santa Cruz sc–33 778; anti–RRP6, GeneTex GTX107856). After two washes in buffer A (5 min each), PLA probes (secondary antibodies conjugated to oligonucleotides) were added and incubated at 37°C for 1 h. Ligation was performed at 37°C for 30 min, followed by amplification at 37°C for 100 min in the dark. Slides were washed with buffer B for 10 min and mounted. All steps were performed in a humidified chamber protected from light. PLA dots per 100 cells were quantified from at least three fields to detect DGCR8 in complex with either RRP6 (the RNA EC, depicted as red signals) or Drosha (the MC, depicted as green signals) in individual patient tumor samples. As absolute PLA signal intensities can vary greatly between tumors from different patients, we quantified these interactions as relative proportions within each patient’s sample rather than comparing absolute signals across different tumors. For each patient, DGCR8/Drosha (MC) and DGCR8/RRP6 (EC) PLA signals were summed to obtain a per–sample total, and MC% = MC/(MC + EC) × 100 and EC% = EC/(MC + EC) × 100 were computed to control for inter–patient variability in absolute signal. The patient samples are arranged along the x-axis in order of increasing HIF-1α protein levels (indicated by the blue line). This proportion-based analysis controlled for inter-patient variability and showed the relationship between HIF-1α levels and DGCR8 complex preference.

### Model organism information

*Caenorhabditis elegans* strains were cultured on nematode growth medium plates seeded with *Escherichia coli* OP50 at 20°C. Populations were synchronized by alkaline hypochlorite treatment of well–fed adults; synchronized larvae were grown on *E. coli* OP50 for 3 days at 20°C to reach day–1 adulthood. Strains used included N2 Bristol, hif–1 (ia4), and ials34 [Phif–1::hif–1(P621G)::myc] (Caenorhabditis Genetics Center, CGC). *D. melanogaster* were reared on standard medium at 25°C under a 12–h light/12–h dark cycle. Gal4 driver lines were crossed to UAS–simaRNAi (P[TRiP.HMS00833]) for genetic studies.

### Quantification and statistical analysis

Data were analyzed using Prism 9 (GraphPad, La Jolla, CA). Results are presented as mean ± SEM from at least three independent experiments. Unpaired two–tailed Student’s *t*–tests were used for two–group comparisons. For multiple groups, one–way analysis of variance with Tukey’s multiple–comparisons test was applied.

## Results

### HIF-1α redirects DGCR8 from microprocessor to RNA exosome and facilitates its nucleoplasmic distribution

**Figure 1.**
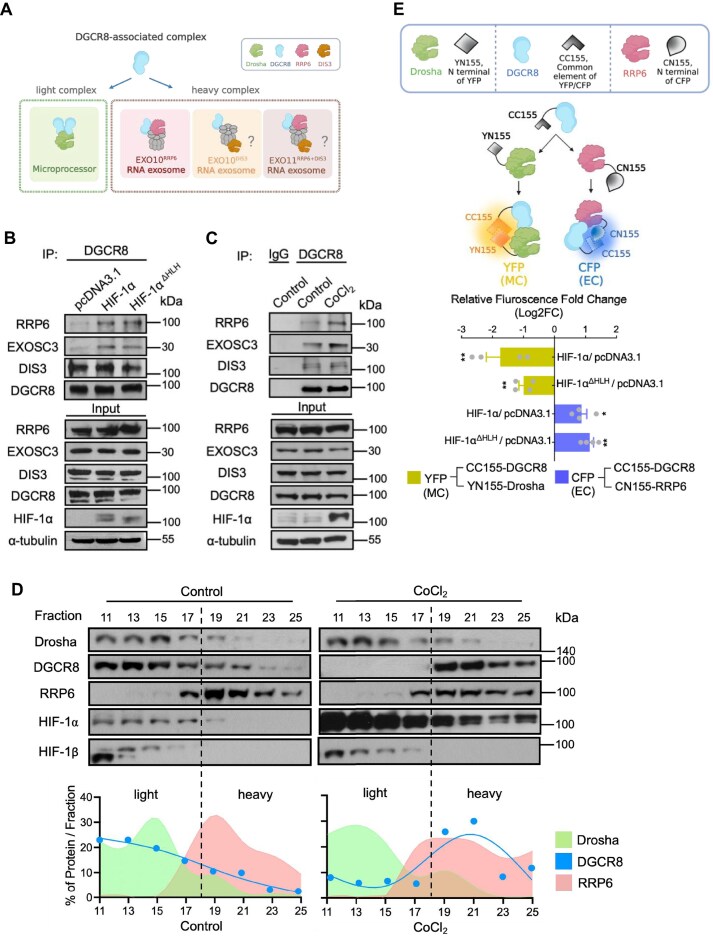
HIF-1α-modulated DGCR8 conversion from microprocessor to RNA Exosome. (**A**) Schematic illustrating the two major DGCR8-containing complexes: the MC and the RNA EC. Endogenous interactions of DGCR8 with RRP6, DIS3, and EXOSC3 in HEK293T cells overexpressing HIF-1α or HIF-1α^ΔHLH^ (**B**), and in HEK293T cells treated with CoCl_2_ to prevent HIF-1α degradation (**C**). Two control conditions were included: the first control consisted of cells treated with ddH_2_O (solvent control for CoCl_2_) and subjected to immunoprecipitation (IP) using an IgG antibody (IP control); the second control involved cells treated with equal amount of ddH_2_O and immunoprecipitated with an anti-DGCR8 antibody. (**D**) Glycerol gradient (30%–50%) sedimentation patterns showing the distribution of endogenous Drosha, DGCR8, and RRP6 in cells treated with CoCl_2_ to prevent HIF-1α degradation. Fractions 11–17 represent the lighter, Drosha-containing complex, and fractions 19–25 indicate the heavier, RRP6-containing complex. (**E**) MiFC assay demonstrating the DGCR8-centered shift from Drosha (YFP signal) to RRP6 (CFP signal) upon HIF-1α or HIF-1α^ΔHLH^ expression. (E, upper panel) Experimental design for the MiFC assay, using chimeric constructs of DGCR8, Drosha, and RRP6. (E, bottom panel) Fluorescence intensities of the YFP (DGCR8/Drosha) and CFP (DGCR8/RRP6) were measured from total protein lysates using a multi-mode microplate reader with specific filter in transfected HEK293T cells. Each data point in the graphs represents the population-averaged fluorescence intensity from independent biological replicates (*n* ≥ 3), with each replicate measured in duplicate. Data are presented as mean ± SEM (*n* ≥ 3), with individual data points shown. *P < 0.05; **P < 0.01.

We recently showed that HIF-1α binds to monomeric DGCR8, thereby inhibiting MC assembly and suppressing primary miRNA biogenesis [10]. Notably, HIF-1α’s ability to interact with DGCR8 in the absence of Drosha prompted us to investigate any MC-independent functions of this newly formed complex [10]. It is now evident that DGCR8 can carry out additional roles by associating with other binding partners, independent of its microprocessor duties [28, 29]. Apart from the MC, DGCR8 is also recognized as an adaptor component of the RNA EC, a large assembly made up of nine core structural proteins and enzymatic subunits RRP6 (EXO10^RRP6^), DIS3 (EXO10^DIS3^), or both (EXO11^RRP6+DIS3^) (Fig. 1A) [11–13, 28]. Since HIF-1α does not alter the expression of these RNA exosome subunits (Supplementary Fig. S1), we asked whether association with HIF-1α reconfigures the binding preferences of DGCR8 and promotes DGCR8 participating in different complexes. Through ectopic overexpression of HIF-1α or using cobalt chloride (CoCl_2_) to inhibit PHD activity, thereby stabilizing HIF-1α by preventing its hydroxylation and degradation [5, 30–33], we induced or stabilized HIF-1α expression to investigate the hypothesis. Following our finding that HIF-1α suppresses microprocessor formation (Supplementary Fig. S2), we observed that HIF-1α increases the binding of DGCR8 to RRP6 and Exosome component 3 (EXOSC3), a core structural protein of the RNA EC, pointing to the formation of an RRP6-containing RNA exosome (EXO10^RRP6^ or EXO11^RRP6+DIS3^) (Fig. 1B and C). Meanwhile, the DGCR8-DIS3 interaction remained unchanged, implying that HIF-1α specifically promotes DGCR8/EXO10^RRP6^ complex formation (Fig. 1B and C). In addition, given that HIF-1α is a well-characterized transcription factor, we further disrupted its DNA-binding ability by truncating the bHLH DNA-binding domain [9, 10]. This HLH-truncated HIF-1α allowed us to investigate whether the transcriptional activity of HIF-1α is required [34–39]. We observed the same effect with HLH-truncated HIF-1α as with full-length HIF-1α, suggesting a transcription-independent function of HIF-1α (Fig. 1B).

To further verify the interaction between DGCR8 and RRP6, the BiFC assay was utilized by designing the two chimeric proteins. VN173-DGCR8 and VC155-RRP6, constructing N-terminal of Venus protein to DGCR8 and C-terminal of Venus protein to RRP6, were used for detecting binding between DGCR8 and RRP6, and our results showed a reconstitutional signal of Venus fluorescence in cells expressing half of Venus-fused DGCR8 and RRP6 (Supplementary Fig. S3). In addition, RNase A was used to determine the RNA dependence of protein binding within the newly formed RNA EC. DGCR8–RRP6 binding remained unaltered, indicating the binding occur in an RNA-independent manner (Supplementary Fig. S4). Density gradient ultracentrifugation further supported that in HIF-1α-accumulated cells under CoCl_2_ treatment, DGCR8 relocates from a light, Drosha-containing complex (fractions 11–17) to a heavy, RRP6-containing complex (fractions 19–25; Fig. 1D). To determine this dynamic MC-to-EC molecular switch, we developed a MiFC assay [40] to monitor the reciprocal interactions among these proteins. Because the YFP and CFP share the same C-terminal sequence (amino acids 156–238) but differ in their N-terminal regions, we fused the common C-terminus to DGCR8 (CC155-DGCR8), the YFP N-terminus to Drosha (YN155-Drosha), and the CFP N-terminus to RRP6 (CN155-RRP6) (Fig. 1E, upper panel). Using this system to simultaneously monitor signals coming from complexes formed by different chimeric fusion proteins, we observed decrease (<0) in log_2_ YFP signal (DGCR8-Drosha) and increase (>0) in log_2_ CFP signal (DGCR8-RRP6) when wild-type or HLH-truncated HIF-1α was overexpressed (Fig. 1E, bottom panel), reflecting a redistribution of DGCR8 binding partners. The same data are also shown as relative fold change normalized to pcDNA3.1 (= 1), where values <1 or >1 correspond to decreased or increased interactions, respectively (Supplementary Fig. S5). Mechanistically, we demonstrated a DGCR8-centered molecular conversion triggered by HIF-1α, from MC to EC (hereinafter refer to as “MC-to-EC switch”). In this event, DGCR8, classically known as a core component of the MC with Drosha, undergoes a functional reassignment upon HIF-1α induction. Specifically, DGCR8 shifted its binding partner from Drosha to the RNA exosome, indicating a regulated switch in complex association. Our recent work showed that HIF-1α binds monomeric DGCR8, thereby disrupting microprocessor formation [10], and here, we found that HIF-1α prompts DGCR8 to associate with the RNA exosome (Fig. 1). In line with this, DGCR8 mutants (DGCR8^W329A^/DGCR8^W329H^), which exist predominantly as monomers, showed enhanced RRP6 binding (Supplementary Fig. S6), further confirming our hypothesis.

**Figure 2.**
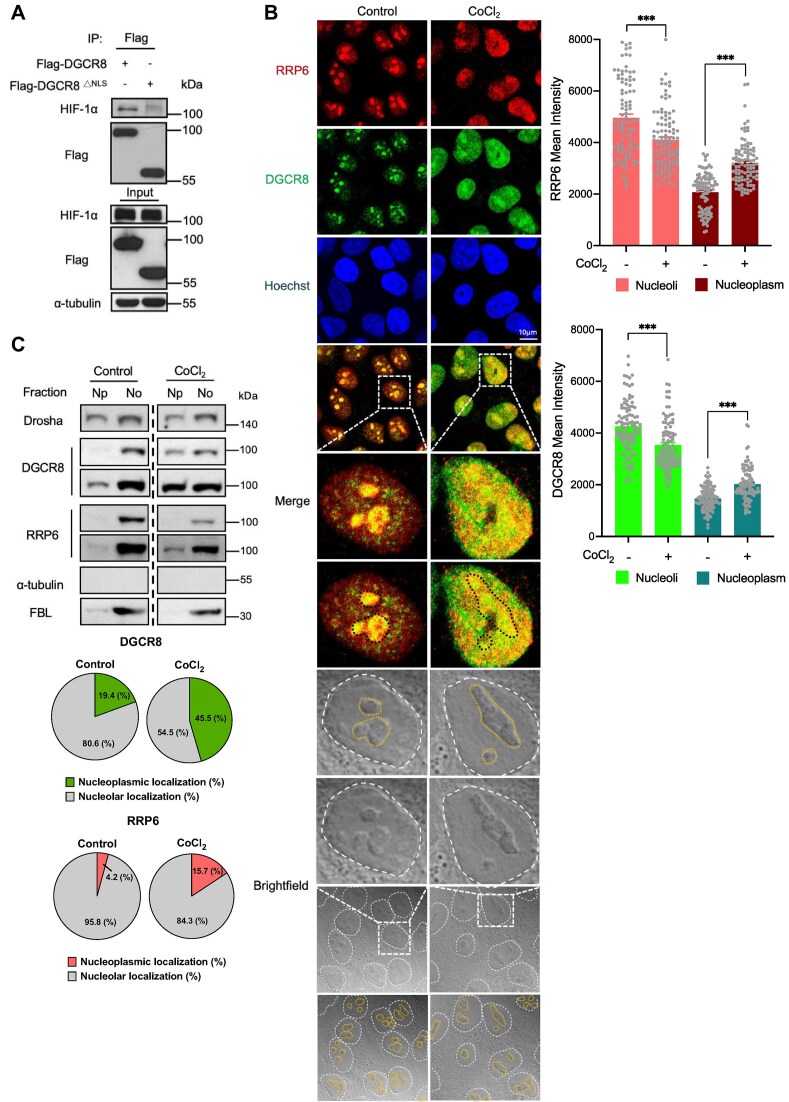
Nucleolar release of DGCR8 and RRP6 under HIF-1α induction. (**A**) Binding between NLS-truncated DGCR8 and HIF-1α was determined by immunoprecipitation. (**B**) Subcellular localizations of endogenous DGCR8 and RRP6 in HIF-1α-accumulated cells under CoCl_2_ treatment, visualized by dual–color immunofluorescence. Nucleoli are identified as round, refractile, phase–dense structures in the brightfield channel [62–65] (outlined in yellow), and the corresponding regions in the fluorescence images are marked with black boundaries. Mean fluorescence intensity in nucleoli and nucleoplasm was quantified for each protein from 100 cells per condition across three independent fields. ***P < 0.001. (**C**) Immunoblot of DGCR8 and RRP6 in subnuclear fractions. α-tubulin serve as a negative control from cytoplasm and FBL (fibrillarin) serve as a positive control for nucleoli. Dashed lines indicate non-adjacent lanes from the same blot processed in parallel.

We next investigated the subcellular localization of this process. Both RRP6 and DGCR8 are known nucleolar proteins [28]. Therefore, by using nuclear localization signal (NLS)-truncated DGCR8, which showed a cytoplasm-retained pattern (Supplementary Fig. S7), we observed a weakened interaction between DGCR8^ΔNLS^ and HIF-1α compared to full-length DGCR8, suggesting the nuclear binding of HIF-1α and DGCR8 (Fig. 2A). A study recently reported a translocation of RRP6 from nucleolus to nucleoplasm under hypoxia [41]. Echoing this observation, our immunostaining revealed that DGCR8 and RRP6, which usually concentrate in nucleoli, are instead released into the nucleoplasm in HIF-1α-accumulated cells under CoCl_2_ treatment (Fig. 2B). This redistribution was further verified using biochemical nucleoli isolation that DGCR8 and RRP6 showed an increased nucleoplasmic distribution in HIF-1α-accumulated cells under CoCl₂ treatment (Fig. 2C). Altogether, these findings demonstrate that the MC-to-EC switch is accompanied by an intranuclear relocation of DGCR8 and RRP6 from the nucleoli to the nucleoplasm by HIF-1α.

### Evolutionary conserved MC-to-EC switch triggered by HIF-1α

**Figure 3.**
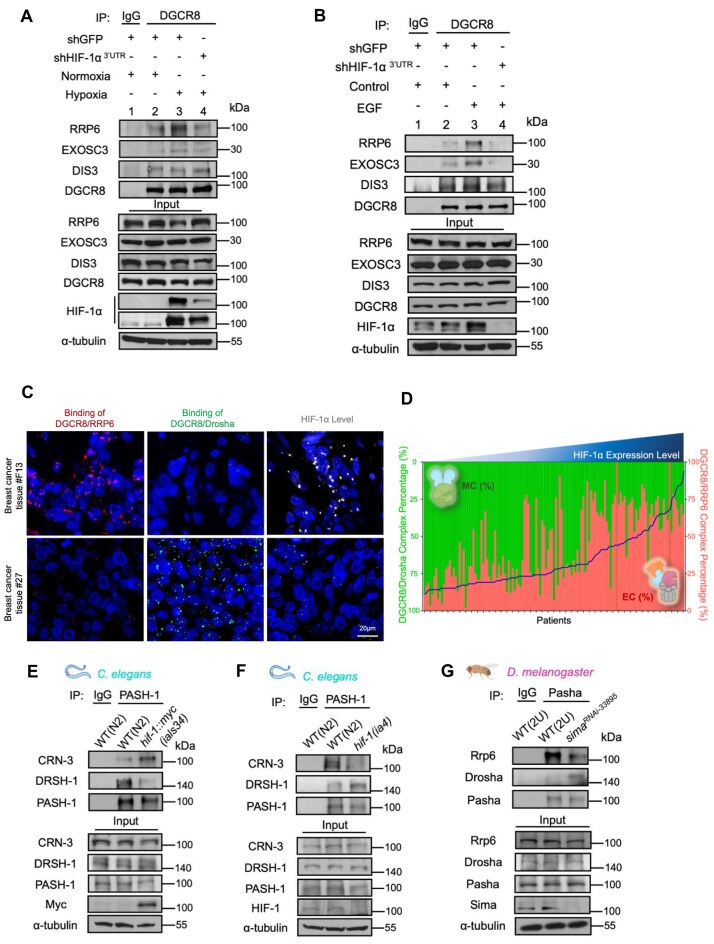
Conservation of the HIF-1α-mediated MC-to-EC switch across species and biological contexts. (**A, B**) Formation of the DGCR8/EXO10RRP6 complex under biological stimuli: hypoxia in MCF-7 cells with HIF-1α knockdown (#819, A), and EGF treatment in MDA-MB-231 cells with HIF-1α knockdown (#819, B). Two control conditions are included: the first control consisted of cells treated with ddH_2_O (solvent control for EGF) and subjected to immunoprecipitation using an IgG antibody (IP control); the second control involved cells treated with equal amount of ddH_2_O and immunoprecipitated with an anti-DGCR8 antibody. (**C**) PLA in human breast cancer tissues (#F13, #27) to detect DGCR8-RRP6 (red) and DGCR8-Drosha (green) complexes. Left, DGCR8/RRP6 proximity (PLA signal, red); middle, DGCR8/Drosha proximity (PLA signal, green); right, HIF-1α level (PLA signal, white). Nuclei are counterstained with DAPI (blue). (**D**) Patient–normalized PLA quantification reveals a shift in DGCR8 complex preference with increasing HIF–1α. For each patient tumor specimen, PLA signals for DGCR8/Drosha (MC, green bars) and DGCR8/RRP6 (EC, red bars) were normalized within-sample and expressed as percentages of the total DGCR8–associated complexes (MC% + EC% = 100%). Patients are ordered along the x–axis by increasing HIF–1α protein level (blue line). (**E, F**) Evidence of MC-to-EC switch in *C. elegans*. Interactions among PASH-1, CRN-3, and DRSH-1 in wild-type (N2), gain-of-function (*ials34*, E), and loss-of-function (*ia4*, F) hif-1 mutants. (**G**) MC-to-EC switch in *D. melanogaster*. Pasha interaction with Rrp6 or Drosha in wild-type (2U) flies and sima-knockdown flies. DRSH-1, HIF-1, PASH-1, and CRN-3 are, respectively, human Drosha, HIF-1α, DGCR8, and RRP6 orthologs in *C.elegans*; Rrp6, Sima, and Pasha are, respectively, human RRP6, HIF-1α, and DGCR8 orthologs in *D. melanogaster*.

To further validate the HIF-1α-mediated association of DGCR8 with the RNA EC in physiological contexts, we examined the assembly of DGCR8/EXO10^RRP6^ under different stimuli. For optimal responsiveness, we used MCF-7 cells for hypoxia experiments due to relatively low basal HIF-1α levels and enabling a clearer and broader dynamic range upon exposure to hypoxia (1% O_2_) (Supplementary Fig. S8A and B). In contrast, MDA-MB-231 cells served as the epidermal growth factor (EGF)-stimulation model because of their high epidermal growth factor receptor (EGFR) expression and more responsive to EGF-induced HIF-1α activation (Supplementary Fig. S8A and C). In cells exposed to hypoxia (Fig. 3A and Supplementary Fig. S9A, lane 3) or EGF (Fig. 3B and Supplementary Fig. S9B, lane 3), formation of DGCR8/EXO10^RRP6^ complex was notably enhanced. However, knocking down HIF-1α substantially reduced the hypoxia- (Fig. 3A and Supplementary Fig. S9A, lane 4) and EGF-induced (Fig. 3B and Supplementary Fig. S9B, lane 4) association of DGCR8 with RRP6, while neither the expression of DGCR8 nor EC components was changed under hypoxic conditions (Supplementary Fig. S10). We extended these observations to clinical samples of human breast cancer by performing a PLA to evaluate the presence of Drosha/DGCR8 (MC; green) and DGCR8/RRP6 (RNA EC; red). In a representative tumor with high HIF-1α expression (breast cancer tissue #F13), the EC signal (red) was markedly increased, whereas the MC signal (green) was diminished. Conversely, in tissue with low HIF-1α expression (breast cancer tissue #27), the opposite pattern was observed (Fig. 3C). We quantified *in situ* PLA signals for DGCR8 interactions with RRP6 (RNA EC; red bars) or Drosha (MC; green bars) in individual patient tumors (Fig. 3D). Each bar represented one patient and showed the relative proportion of DGCR8-associated EC versus MC interactions. Samples are ordered along the x-axis by increasing HIF-1α protein level (blue line). This presentation revealed a graded, reciprocal pattern across the patient series, as HIF-1α (blue line) increased from left to right, EC% (red) progressively rose, while MC% (green) declined. The pattern, MC-dominant at low HIF-1α to EC-dominant at high HIF-1α, demonstrated the HIF-1α-mediated MC-to-EC shift, which is consistent with a redistribution of DGCR8 from the microprocessor to the EC (Fig. 3D). To test whether this MC-to-EC switch is evolutionarily conserved, we analyzed model organisms. In *C. elegans*, where HIF-1 is the ortholog of human HIF-1α, a gain-of-function (*ials34)* mutant [42] consistently showed elevated PASH-1/CRN-3 (PASH-1 and CRN-3 being orthologs of DGCR8 and RRP6, respectively) alongside reduced PASH-1/DRSH-1 (the *C. elegans* Drosha ortholog) (Fig. 3E). Conversely, a loss-of-function *hif-1* mutant (*ia4*) [42] exhibited heightened PASH-1/DRSH-1 binding and diminished PASH-1/CRN-3 association (Fig. 3F). Likewise, in *D. melanogaster*, knockdown of *sima* (the HIF-1α ortholog) led to reduced interactions between Pasha (DGCR8 ortholog) and Rrp6 (the RRP6 ortholog), accompanied by a reciprocal increase in Pasha/Drosha binding (Fig. 3G). These results collectively confirm that the HIF-1α-driven MC-to-EC switch is evolutionarily conserved across diverse species, ranging from *C. elegans* and *D. melanogaster* to humans, and across different biological contexts such as genetic manipulations, hypoxia, and growth factor stimulation.

### HIF-1α facilitates EC-mediated snoRNA degradation

**Figure 4.**
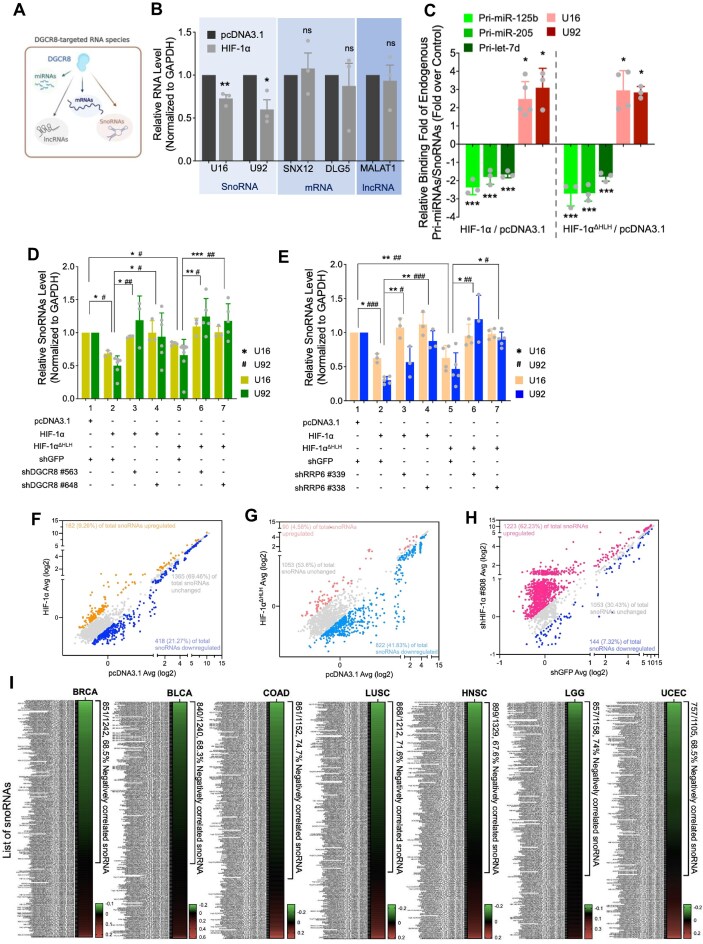
HIF-1α-driven MC-to-EC switch promotes snoRNA downregulation. (**A**) Schematic representation of RNA species associated with DGCR8. (**B**) Level of snoRNAs (U16, U92), mRNAs (SNX12, DLG5), and the lncRNA MALAT1 in HEK293T cells expressing HIF-1α. (**C**) RIP with an anti-DGCR8 antibody in HEK293T cells expressing HIF-1α or HIF-1α^ΔHLH,^ followed by qPCR detecting pri-miRNAs and snoRNAs. (D, E) Effects of DGCR8 or RRP6 knockdown on HIF-1α-regulated snoRNAs. Level of U16 and U92 in HIF-1α- and HIF-1α^ΔHLH^-expressing HEK293T cells upon DGCR8 knockdown (**D**) or RRP6 knockdown (**E**). Data in panels (B–E) are from independent biological repeats and shown as mean ± SEM (*n* ≥ 3), with individual data points. */#*P *< 0.05, **/# #*P *< 0.01, ***/###*P *< 0.001. *Represents statistical significance of U16; #represents statistical significance of U92. SnoRNA profiling in MCF-7 cells expressing wild-type HIF-1α (**F**) or HLH-truncated HIF-1α (**G**), depicted as scatter plots. Orange or red dots represent upregulated snoRNAs, blue dots represent downregulated snoRNAs, gray dots represent unchanged snoRNAs based on the fold change cutoff of 1.2. (**H**) SnoRNA profile in MDA-MB-231 cells with HIF-1α knockdown. Red dots represent upregulated snoRNAs, blue dots represent downregulated snoRNAs, and gray dots represent unchanged snoRNAs based on the fold change cutoff of 1.2. Microarray profiling was performed using the GeneChip™ miRNA 4.0 Array platform (Applied Biosystems™, #902411). The microarray dataset was deposited in GEO under accession number GSE313594. (**I**) Heatmaps show Pearson correlation coefficients (*r*) between HIF1A mRNA and all annotated snoRNAs across different tumor types (BRCA, BLCA, COAD, LUSC, HNSC, LGG, UCEC). HIF-1α expression was quantified as *Z*-scores, and snoRNA expression as TPM. Color scales indicate r per snoRNA (rows). Percentages denote the fraction of snoRNAs with *r* < 0 in each cohort showing the negative correlations. Abbreviations: BRCA, breast invasive carcinoma; BLCA, bladder urothelial carcinoma; COAD, colon adenocarcinoma; LUSC, lung squamous cell carcinoma; HNSC, head and neck squamous cell carcinoma; LGG, lower-grade glioma; and UCEC, uterine corpus endometrial carcinoma.

Given that DGCR8 acts as a versatile RNA-binding protein that targets various RNA species for RNA processing or downregulation [29] (Fig. 4A), we assessed whether HIF-1α could influence DGCR8-targeted RNAs, including miRNAs, mRNAs, lncRNAs, and snoRNAs. Of these candidates, only snoRNAs U16 and U92, representing the two major classes of snoRNAs (C/D box and H/ACA box, respectively), were consistently and significantly downregulated by HIF-1α (Fig. 4B). In contrast, the tested mRNAs (SNX12 and DLG5) and the lncRNA (MALAT1) remained unchanged (Fig. 4B). In parallel, RIP experiments revealed a shift in DGCR8’s RNA-binding preference when wild-type or HLH-truncated HIF-1α was expressed. Specifically, association of DGCR8 with pri-miRNAs was reduced (Fig. 4C, green bars), while binding to snoRNAs was increased (Fig. 4C, red bars) under the expression of wild-type or HLH-truncated HIF-1α. These observations indicated that snoRNAs serve as substrates for the newly formed EC. Next, knockdown of either DGCR8 or RRP6 abrogated HIF-1α-mediated downregulation of snoRNAs U16 and U92 (Fig. 4D and E), confirming that the RNA EC is essential for HIF-1α-mediated snoRNA downregulation.

To determine the genome-wide effects of HIF-1α on snoRNA level, we employed the GeneChip™ miRNA 4.0 Array platform, which includes 1965 snoRNAs among 39 505 total targets (Applied Biosystems™, #902411). Our results showed that 9.26% (182/1965) of differentially expressed snoRNAs were upregulated in wild-type HIF-1α-expressing cells (Fig. 4F and Supplementary Table S3), whereas 21.27% (418/1965) were downregulated under the same condition (Fig. 4F and Supplementary Table S3). This reduction became even more marked in cells expressing HLH-truncated HIF-1α, where 41.38% (822/1965) of differentially expressed snoRNAs were decreased relative to controls (Fig. 4G and Supplementary Table S3). Conversely, HIF-1α knockdown significantly elevated global snoRNA level, with 62.23% (1223/1965) of differentially expressed snoRNAs displaying increased levels (Fig. 4H). Further aligning with our previous reports linking high HIF-1α expression to reduced miRNA levels [9, 10], our analysis of the Cancer Genome Atlas datasets revealed that the majority of snoRNAs displayed negative correlations with HIF-1α levels in different cancer types (67.6%–74.7%) (Fig. 4I). Collectively, these data suggested the broad impact of HIF-1α on snoRNA downregulation across diverse cancer contexts.

**Figure 5.**
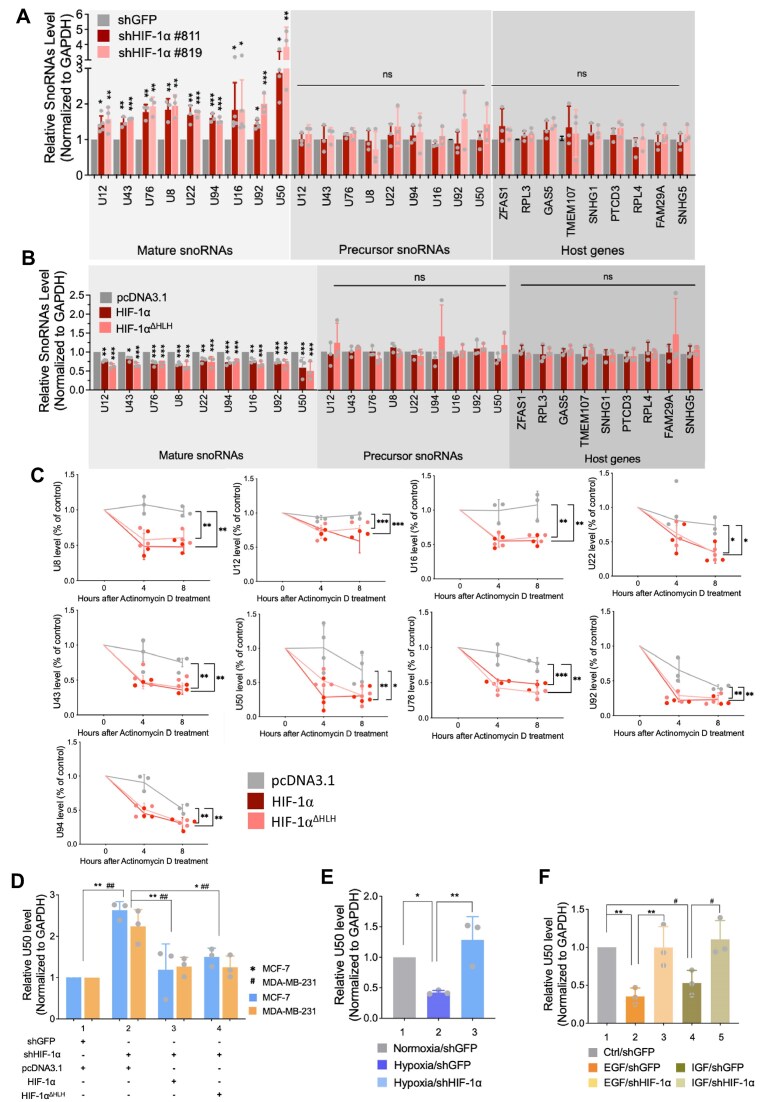
Effects of HIF-1α on snoRNA biosynthesis and turnover. Levels of snoRNAs, precursors, and host genes in MDA-MB-231 cells with HIF-1α knockdown (**A**) and in MCF-7 with HIF-1α overexpression (**B**). (**C**) SnoRNAs stability assay in MCF-7 cells expressing HIF-1α or HIF-1α^ΔHLH^, as measured by actinomycin D treatment. Actinomycin D (5 µg ml^−1^) was then treated, and RNA was collected at 0, 4, and 8 h. The indicated snoRNAs were quantified by RT-qPCR plotted as % compared to 0 h. (**D**) Level of U50 after HIF-1α restoration in MCF-7 and MDA-MB-231 cells with HIF-1α knockdown. Validation of HIF-1α-mediated U50 downregulation under various stimuli: hypoxia in MCF-7 cells (**E**) and EGF/IGF treatment in MDA-MB-231 cells (**F**). Data are from independent biological repeats and shown as mean ± SEM (*n* ≥ 3), with individual data points. */#*P *< 0.05, **/# #*P *< 0.01, ***/###*P* < 0.001. *Represents statistical significance in MCF-7 cells; #represents statistical significance in MDA-MB-231 cells (E). *Represents statistical significance of EGF treatment; #represents statistical significance of IGF treatment (F).

Although HIF-1α broadly suppresses snoRNA level, the snoRNAs differentially regulated by HIF-1α showed no noticeable bias toward either C/D box or H/ACA box classes (Supplementary Fig. S11). While snoRNAs are classically regarded as housekeeping genes involved in directing rRNA modifications to maintain cellular homeostasis, recent studies have uncovered additional roles for certain snoRNAs (U12, U43, U76, U50, U8, U22, U16, U92, and U94) in pathological processes [43–47]. Given our evidence that HIF-1α triggers an MC-to-EC switch that shifts DGCR8’s substrate preference from pri-miRNAs to snoRNAs, resulting in snoRNA downregulation, we next assessed the expression profiles of these snoRNAs from their host genes and precursors to their mature forms. Notably, only the mature snoRNAs were broadly elevated in HIF-1α knockdown cells (Fig. 5A and Supplementary Fig. S12) and downregulated in wild-type or HLH-truncated HIF-1α overexpressing cells (Fig. 5B), while neither their precursors nor host genes exhibited significant changes (Fig. 5A and B). To realize how HIF-1α downregulates snoRNAs, other than altering transcription or maturation, we used actinomycin D assay to investigate the effect of HIF-1α on snoRNAs stability. Half-life of snoRNAs were decreased in cells expressing either wild-type or HLH-truncated HIF-1α, indicating the enhanced snoRNA degradation (Fig. 5C). Following the conclusion that HIF-1α downregulates snoRNAs through RNA exosome (Fig. 4D and E), directly suppressing RRP6 expression by RRP6 knockdown or inhibiting RRP6 activity by 5-FU treatment was strategically used to verify the RNA exosome dependence in HIF-1α-mediated snoRNAs turnover [48–50]. Supportively, HIF-1α-induced snoRNA degradation was reversed upon RRP6 knockdown or 5-FU treatment (Supplementary Fig. S13). Because steady-state RNA abundance reflects the ratio of synthesis to decay, the unchanged synthesis coupled with an increased decay indicates that degradation is the driving force behind the observed downregulation. Among the snoRNAs examined, U50 was the most sensitive to HIF-1α (Fig. 5A and B; Supplementary Fig. S12), and the increase in U50 level observed upon HIF-1α knockdown (Fig. 5D, lane 2) was diminished by reintroducing HIF-1α or HIF-1α^ΔHLH^ (Fig. 5D, lanes 3–4). Moreover, U50 downregulation induced by hypoxia, EGF, or IGF (Fig. 5E, lane 2 and 5F, lanes 2 and 4) was abrogated when HIF-1α was depleted (Fig. 5E, lane 3 and 5F, lanes 3 and 5). Together, these results support the conclusion that HIF-1α downregulates mature snoRNAs primarily by promoting their degradation through the RNA exosome, rather than by affecting their transcription or maturation.

### HIF-1α-driven snoRNA degradation impairs rRNA modifications and translation efficiency

**Figure 6.**
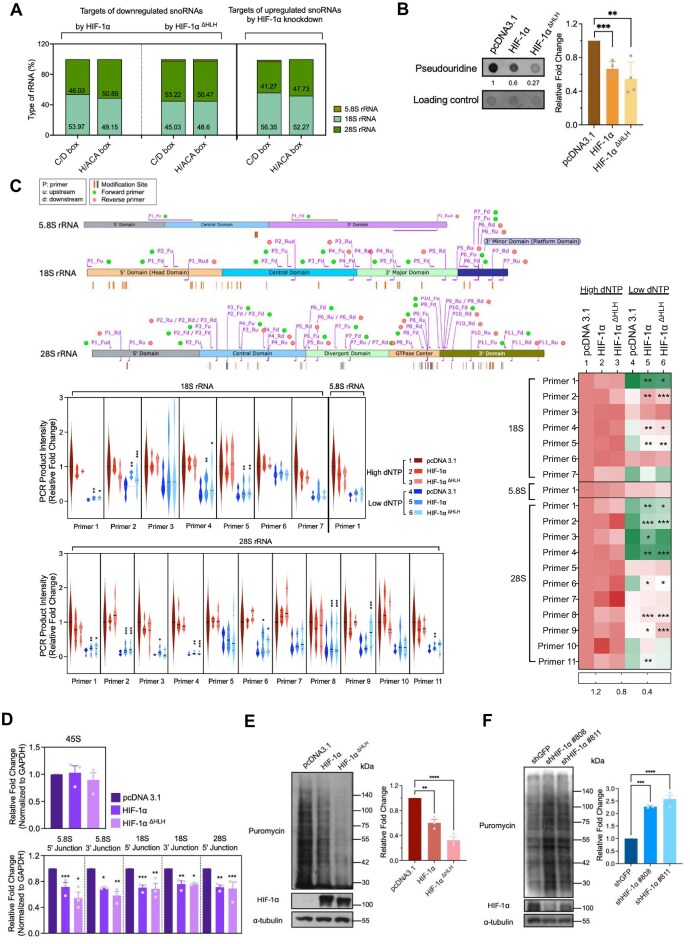
HIF-1α suppresses rRNA modifications and translation efficiency. (**A**) Distribution of rRNAs targeted by snoRNAs differentially expressed under HIF-1α manipulation. (**B**) RNA dot blot revealing pseudouridylation status in HEK293T cells overexpressing HIF-1α or HIF-1α^ΔHLH,^ with Sybr Gold serving as a loading control for total RNA detection. (**C**) RTL-P assay to determine 2′-O-methylation status in 5.8S, 18S, and 28S rRNAs. Blue violin plots/heatmap (lines 4–6) indicate low dNTP conditions; red violin plots/heatmap (lines 1–3) indicate high dNTP conditions. 2′-O-methylated sites were detected by RTL-P and PCR product were quantified by ImageJ. (**D**) qPCR quantification of rRNA precursors (45S) and intermediates, normalized to GAPDH. (**E**) Translation efficiency was detected using anti-puromycin antibody in HIF-1α- and HIF-1αΔHLH-expressing HEK293T cells. (**F**) Translation efficiency was detected using anti-puromycin antibody in HIF-1α knockdown MDA-MB-231 cells. Data are from independent biological repeats and shown as mean ± SEM (*n* ≥ 3), with individual data points. *P < 0.05; **P < 0.01; ***P < 0.001.

snoRNAs serve as critical modifiers of rRNA, including 5.8S, 18S, and 28S rRNAs. However, the snoRNAs differentially regulated by HIF-1α did not show preferential targeting of any single rRNA species (Fig. 6A). Since C/D box and H/ACA box snoRNAs direct 2′-O-methylation and pseudouridylation, respectively [51, 52], we evaluated these modifications under conditions of HIF-1α overexpression. RNA dot blot analysis using anti-pseudouridine antibody revealed a decreased signal in cells expressing either wild-type or HLH-truncated HIF-1α, indicating that HIF-1α suppresses pseudouridylation (Fig. 6B) [53]. To investigate 2′-O-methylation status, we employed the Reverse Transcription at Low dNTP concentrations followed by polymerase chain reaction (RTL-P assay) [22, 53–55], which is a technique specifically designed for site-specific detection of 2′O-methylation. It relies on the principle that 2′O-methylated nucleotides hinder reverse transcription (RT), causing reverse transcriptase to pause and terminate cDNA synthesis at the 2′O-methylation sites under low dNTP concentrations [22, 54, 55]. To this end, we designed primer sets targeting sites with a modification frequency exceeding 80% within rRNAs [56] (Fig. 6C, upper panel). Under low dNTP conditions (Fig. 6C, blue violin plot groups; heatmap, lines 4–6), RT pausing resulted in decreased PCR product intensity compared to high dNTP conditions (Fig. 6C, red violin plot groups; heatmap, lines 1–3). Notably, within the low dNTP group (Fig. 6C, blue groups of violin plot groups; heatmap, line 4–6), PCR product intensity increased in cells expressing either wild-type or HLH-truncated HIF-1α (Fig. 6C, line 5–6 of violin plot groups and heatmap) compared to control group (Fig. 6C, line 4 of violin plot groups and heatmap). This suggested that the enhanced reverse transcription efficiency results from reduced 2′-O-methylation under HIF-1α overexpression. Because rRNA biogenesis is closely linked to rRNA modifications, which influences rRNA processing [57–59], we determined the level of rRNA precursor (45S) and its intermediates [14]. Although no significant change of 45S was observed (Fig. 6D, upper panel), the decreased levels of rRNA intermediates (Fig. 6D, bottom panel) in cells expressing either wild-type or HLH-truncated HIF-1α supported the hypothesis that HIF-1α-mediated downregulation of snoRNAs leads to impaired rRNA biogenesis. It has been reported that rRNA modifications in specific sites within rRNA enhance the translation rate [58, 60]. Therefore, we investigated whether HIF-1α-mediated hypo-modified rRNAs impact translation efficiency by surface sensing of translation assay [61]. Using an anti-puromycin antibody to detect puromycin incorporation, we observed reduced signals, indicating decreased translation efficiency in cells expressing either wild-type or HLH-truncated HIF-1α (Fig. 6E). In contrast, cells lacking HIF-1α showed elevated puromycin signals, reflecting enhanced translation efficiency (Fig. 6F).

In summary, our findings uncover a non-transcriptional function of HIF-1α, whereby HIF-1α-bound DGCR8 shifts its association from the MC to the RNA exosome. This transition drives snoRNA downregulation, leading to hypo-modified rRNAs and impaired translation efficiency.

## Discussion

The RNA exosome is well established as a central hub for RNA turnover, maturation, and surveillance, yet how its activity adapts to diverse biological cues remains unclear. Here, we show that HIF-1α, known primarily as a transcription factor for hypoxic responses, exerts a non-transcriptional function in regulating RNA exosome activity. Specifically, HIF-1α triggers an “MC-to-EC switch,” redirecting DGCR8 from its canonical partner, Drosha (MC), to RRP6 (RNA EC). This discovery not only illuminates a novel facet of DGCR8’s functionality beyond miRNA biogenesis but also broadens our understanding of how stress-related signals recalibrate fundamental RNA-processing machinery. DGCR8 is classically recognized for its role in miRNA biogenesis, yet high-throughput mapping (e.g. HITS-CLIP) has shown that DGCR8 can also bind and regulate mRNAs, lncRNAs, snoRNAs, and telomerase RNA in Drosha-dependent and -independent modes. Our data uncover an additional dimension of DGCR8’s functionality: under the influence of HIF-1α, DGCR8 is diverted from the Microprocessor to the RNA exosome, switching its primary substrate preference from pri-miRNAs to snoRNAs. This pivot not only suppresses pri-miRNA processing but also promotes widespread snoRNA degradation, revealing a dual impact of HIF-1α on distinct classes of noncoding RNA. Notably, other DGCR8-associated RNAs (e.g. mRNAs, lncRNAs) showed no significant changes under HIF-1α induction, indicating that the MC-to-EC switch selectively targets snoRNA stability, although context-dependent effects on additional RNA substrates cannot be ruled out.

Mechanistically, HIF-1α binds DGCR8 by selectively enhancing its interaction with RRP6, forming either EXO10^RRP6^ or EXO11^RRP6+DIS3^, while displacing it from Drosha-containing complexes. Consistent with this partitioning model, glycerol-gradient fractionation revealed that under steady-state conditions, endogenous HIF-1α was primarily detected in the light glycerol-gradient fractions together with HIF-1β (Fig. 1D). Upon CoCl_2_ treatment, HIF-1α accumulated and became detectable across both light and heavy fractions, while its presence with HIF-1β in the light fractions was maintained (Fig. 1D). This distribution suggests that stabilized HIF-1α partitions between canonical HIF-1β-containing complexes and additional high-molecular-weight assemblies in the heavy fractions, consistent with a putative HIF-1α-DGCR8-RNA EC. This reveals a means by which exosome composition can be dynamically reconfigured in response to environmental or growth factor cues. Depletion of snoRNAs through this MC-to-EC switch disrupts critical rRNA modifications, including pseudouridylation and 2′-O-methylation, compromising rRNA processing and ultimately reducing translation efficiency. Importantly, this effect occurs without altering snoRNA transcription or precursor maturation, suggesting a strictly post-transcriptional mechanism that governs snoRNA stability under conditions of HIF-1α induction. The MC-to-EC switch is evolutionarily conserved: we observe the same DGCR8 shift in *C. elegans* and *D. melanogaster*, and human tumors with inverse correlation between HIF-1α and snoRNA levels. Although HIF-1α is traditionally linked to hypoxia, we also find that growth-factor signaling can trigger this program, underscoring the MC-to-EC switch as a versatile adaptive module by which cells tune rRNA biogenesis and translation in rapidly changing microenvironments. Lastly, these results expand DGCR8’s placement in RNA biology. Typically viewed as a Microprocessor component, DGCR8 emerges here as a molecular node that can switch tasks according to cellular context under HIF-1α control. This context-dependent transition between miRNA processing and snoRNA degradation adds a layer of post-transcriptional regulation that helps coordinate global RNA metabolism under stress or developmental cues.

From a physiological perspective, the MC-to-EC switch provides a coherent adaptive strategy to hypoxic and metabolically demanding conditions. Protein synthesis is among the most ATP- and oxygen-consuming processes; reducing ribosome output conserves energy and limits proteotoxic stress when energy supply is limited. Consistent with this view, HIF-1α-driven snoRNA depletion is accompanied by decreased rRNA modification, reduced accumulation of rRNA processing intermediates, and a drop in nascent peptide synthesis. These changes reflect a “slowdown” of translational machinery rather than a complete shutoff. Such graded control supports proteostasis and survival under hypoxia by aligning biosynthetic load with available resources, while preserving the option for rapid recovery once stress resolves. Importantly, the MC-to-EC switch is not restricted to low oxygen. We observe that mitogenic cues (EGF/IGF) can also induce HIF-1α, trigger DGCR8-RRP6 assembly, promote snoRNA degradation, and suppress translation. This seemingly paradoxical outcome suggests a checkpoint that decouples proliferative signaling from translational capacity when anabolic drive risks outstripping metabolic supply. In this model, growth factor-induced HIF-1α tempers translational machinery through snoRNA loss to prevent futile or error-prone translation, thereby safeguarding proteome quality during rapid signaling transitions and conserving energy when energy supply is limited. Such context-dependent translational restraint may be a broader homeostatic principle that limits biosynthetic overshoot under fluctuating nutrient and oxygen availability.

Classically, HIF-1α functions as an oxygen-regulated transcription factor that binds hypoxia-response elements to activate target genes, thereby increasing their mRNA abundance. By contrast, canonical miRNA activity suppresses target gene expression post-transcriptionally, lowering mRNA levels. These axes predict opposite effects on mRNA output. Our prior work [10] revealed a transcription-independent arm whereby HIF-1α disrupts Microprocessor assembly in the nucleus, and together with JCI evidence that HIF-1α dampens Dicer-mediated processing in the cytoplasm [9], establishes that HIF-1α reduces miRNA biogenesis at both nuclear and cytoplasmic steps. The resulting global decrease in mature miRNAs relieves repression of HIF-induced transcripts, producing coordinated derepression. In parallel, this study uncovers a second non-transcriptional program: HIF-1α redirects DGCR8 to an RRP6-containing RNA exosome, promoting snoRNA degradation, diminishing rRNA 2′-O-methylation and pseudouridylation, and slowing rRNA processing and ribosome output. Together, these findings position HIF-1α as a systems-level coordinator: its transcriptional effect increases adaptive mRNA levels; its miRNA effect derepresses them; and its snoRNA/rRNA effect tempers translation to match biosynthetic capacity [9, 10]. In this way, the non-transcriptional mechanisms work together with the transcriptional program, producing a synergistic, resource-aware increase in effective gene expression.

In summary, our study reveals that HIF-1α, beyond its canonical role as an oxygen-regulated transcription factor, orchestrates a transcription-independent program that reshapes post-transcriptional [10] and translational control (this study). Under such a scenario, HIF-1α suppresses miRNA biogenesis thereby relieving repression of stress-responsive transcripts. In parallel, it redirects DGCR8 from the Microprocessor to an RRP6-containing RNA exosome, promoting snoRNA degradation and slowing translational output. Through this mechanism, HIF-1s aligns RNA metabolism with cellular energetic demands, conserving resources under stress or stimulation. Overall, these findings highlight a sophisticated mechanism by which HIF-1α links hypoxic and growth factor signals to core RNA-processing pathways. They also raise the possibility that other signaling pathways might similarly target DGCR8 or the RNA exosome to fine-tune RNA stability and processing, pointing out the need to explore how dysregulation of this MC-to-EC switch contributes to disease.

## Supplementary Material

gkag070_Supplemental_Files

## Data Availability

The microarray dataset has been deposited in GEO under accession number GSE313594.
