# Supplementary material for: A non-canonical role for HIF-1α: redirecting DGCR8 to the RNA exosome for snoRNA degradation and translational modulation
Source: Nucleic Acids Res. 2026 Feb 2;54(3):gkag070. doi: 10.1093/nar/gkag070 (PMC12862392; doi:10.1093/nar/gkag070)
Supplement: gkag070_Supplemental_Files [file gkag070_supplemental_files.zip › Revised Supplementary Figures_20260129_clean.pptx]

## Slide 1
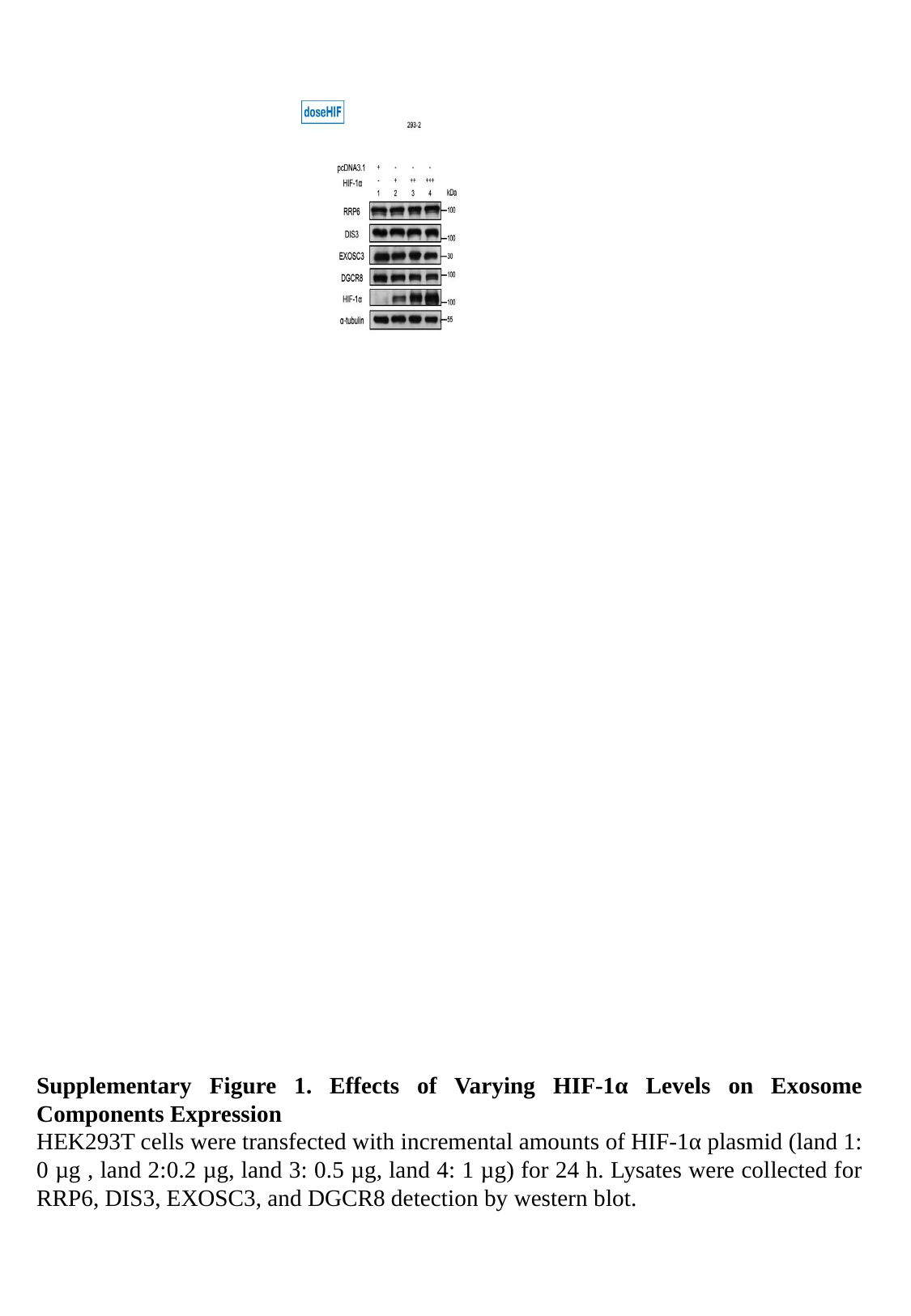

Supplementary Figure 1. Effects of Varying HIF-1α Levels on Exosome Components Expression
HEK293T cells were transfected with incremental amounts of HIF-1α plasmid (land 1: 0 µg , land 2:0.2 µg, land 3: 0.5 µg, land 4: 1 µg) for 24 h. Lysates were collected for RRP6, DIS3, EXOSC3, and DGCR8 detection by western blot.

## Slide 2
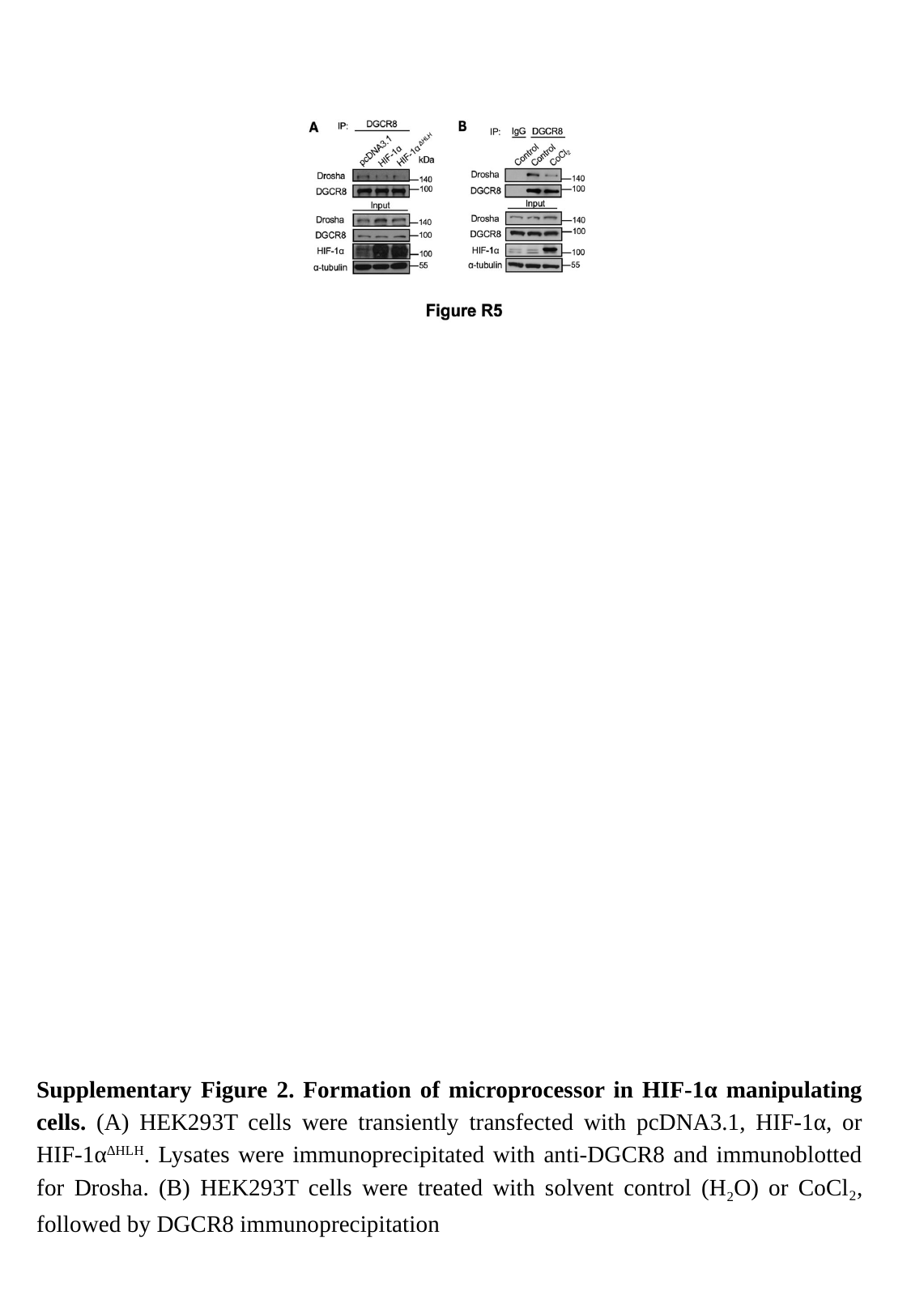

Supplementary Figure 2. Formation of microprocessor in HIF-1α manipulating cells. (A) HEK293T cells were transiently transfected with pcDNA3.1, HIF-1α, or HIF-1αΔHLH. Lysates were immunoprecipitated with anti-DGCR8 and immunoblotted for Drosha. (B) HEK293T cells were treated with solvent control (H2O) or CoCl₂, followed by DGCR8 immunoprecipitation

## Slide 3
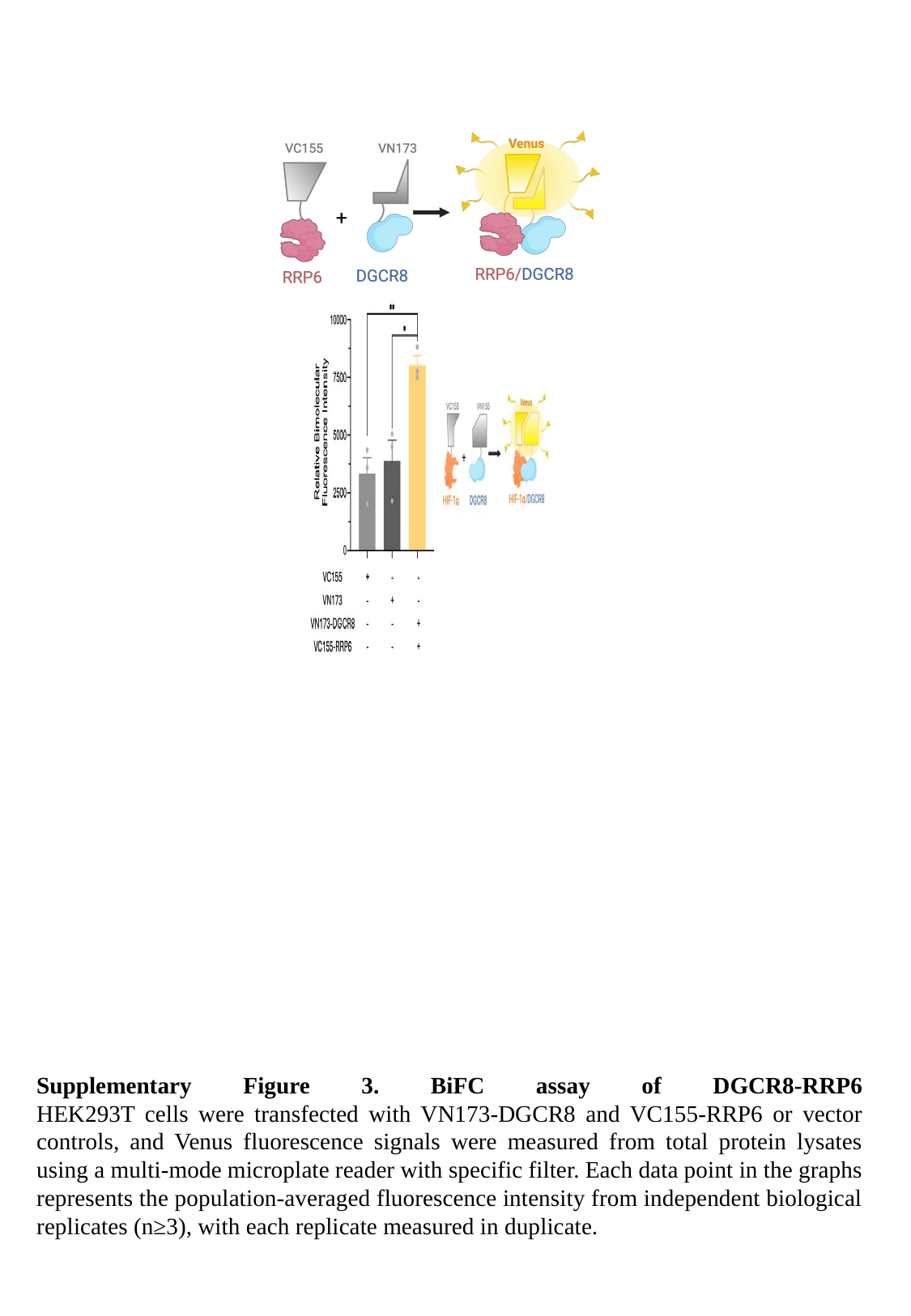

Supplementary Figure 3. BiFC assay of DGCR8-RRP6
HEK293T cells were transfected with VN173-DGCR8 and VC155-RRP6 or vector controls, and Venus fluorescence signals were measured from total protein lysates using a multi-mode microplate reader with specific filter. Each data point in the graphs represents the population-averaged fluorescence intensity from independent biological replicates (n≥3), with each replicate measured in duplicate.

## Slide 4
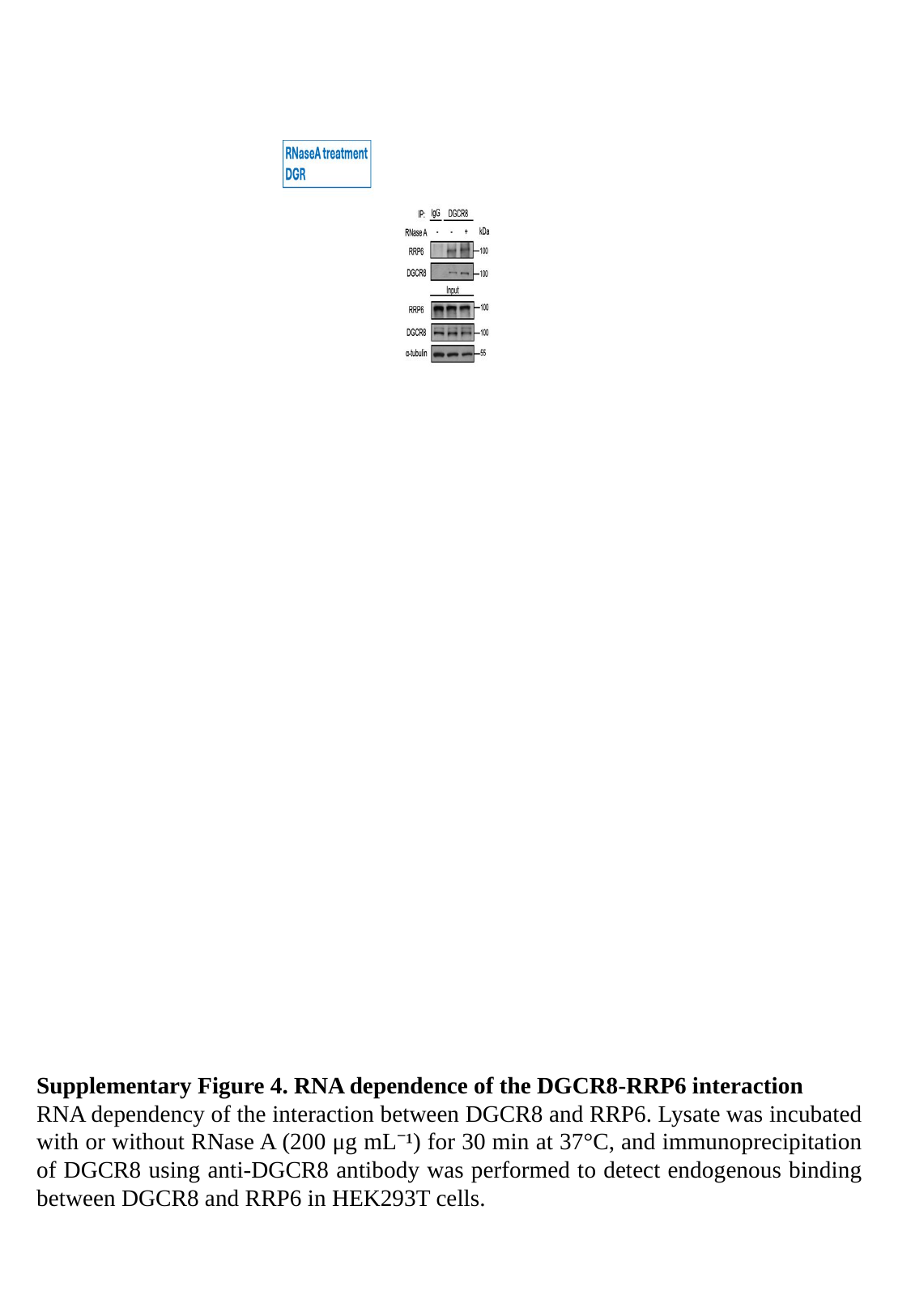

Supplementary Figure 4. RNA dependence of the DGCR8-RRP6 interaction
RNA dependency of the interaction between DGCR8 and RRP6. Lysate was incubated with or without RNase A (200 μg mL⁻¹) for 30 min at 37°C, and immunoprecipitation of DGCR8 using anti-DGCR8 antibody was performed to detect endogenous binding between DGCR8 and RRP6 in HEK293T cells.

## Slide 5
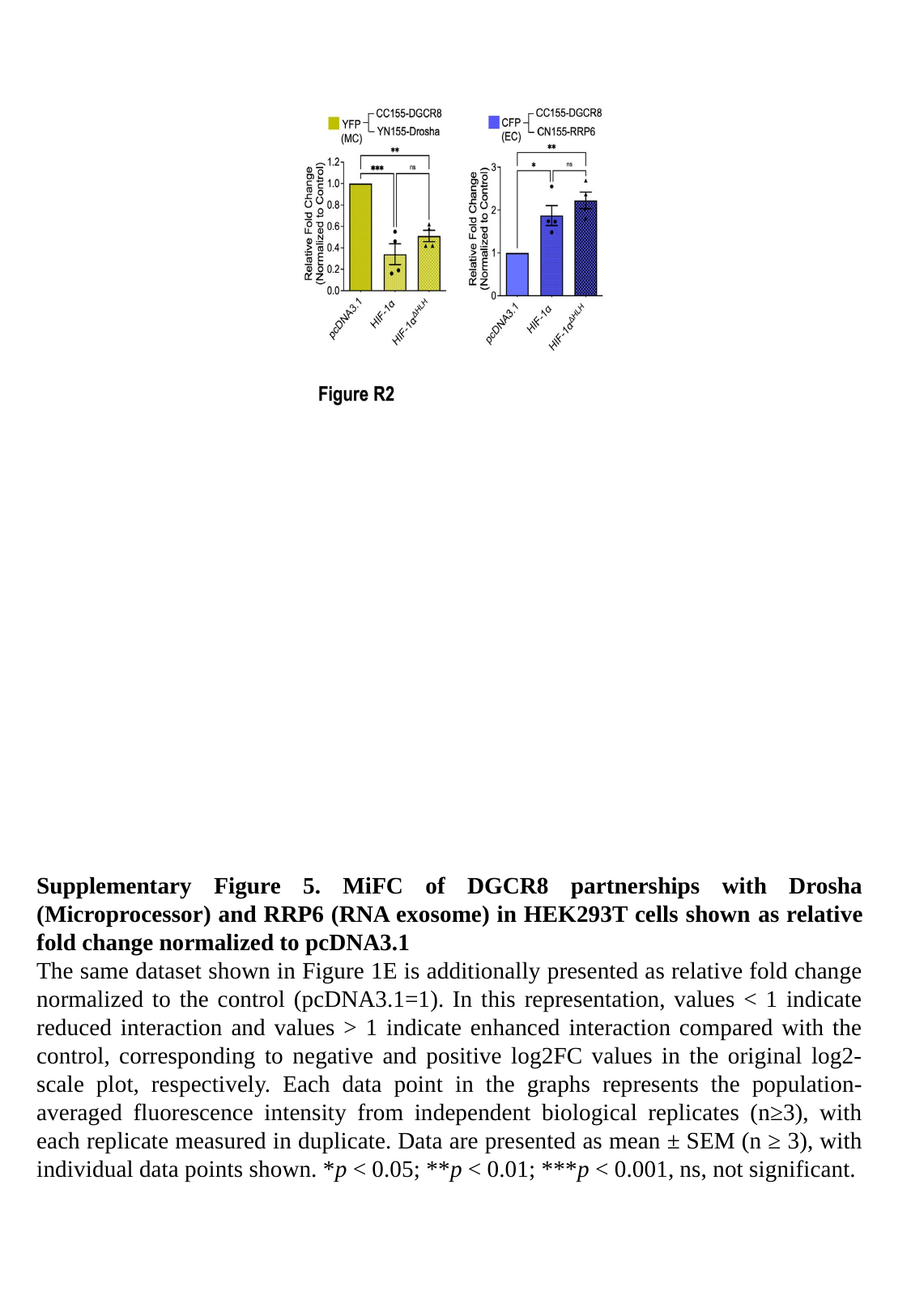

Supplementary Figure 5. MiFC of DGCR8 partnerships with Drosha (Microprocessor) and RRP6 (RNA exosome) in HEK293T cells shown as relative fold change normalized to pcDNA3.1
The same dataset shown in Figure 1E is additionally presented as relative fold change normalized to the control (pcDNA3.1=1). In this representation, values < 1 indicate reduced interaction and values > 1 indicate enhanced interaction compared with the control, corresponding to negative and positive log2FC values in the original log2-scale plot, respectively. Each data point in the graphs represents the population-averaged fluorescence intensity from independent biological replicates (n≥3), with each replicate measured in duplicate. Data are presented as mean ± SEM (n ≥ 3), with individual data points shown. *p < 0.05; **p < 0.01; ***p < 0.001, ns, not significant.

## Slide 6
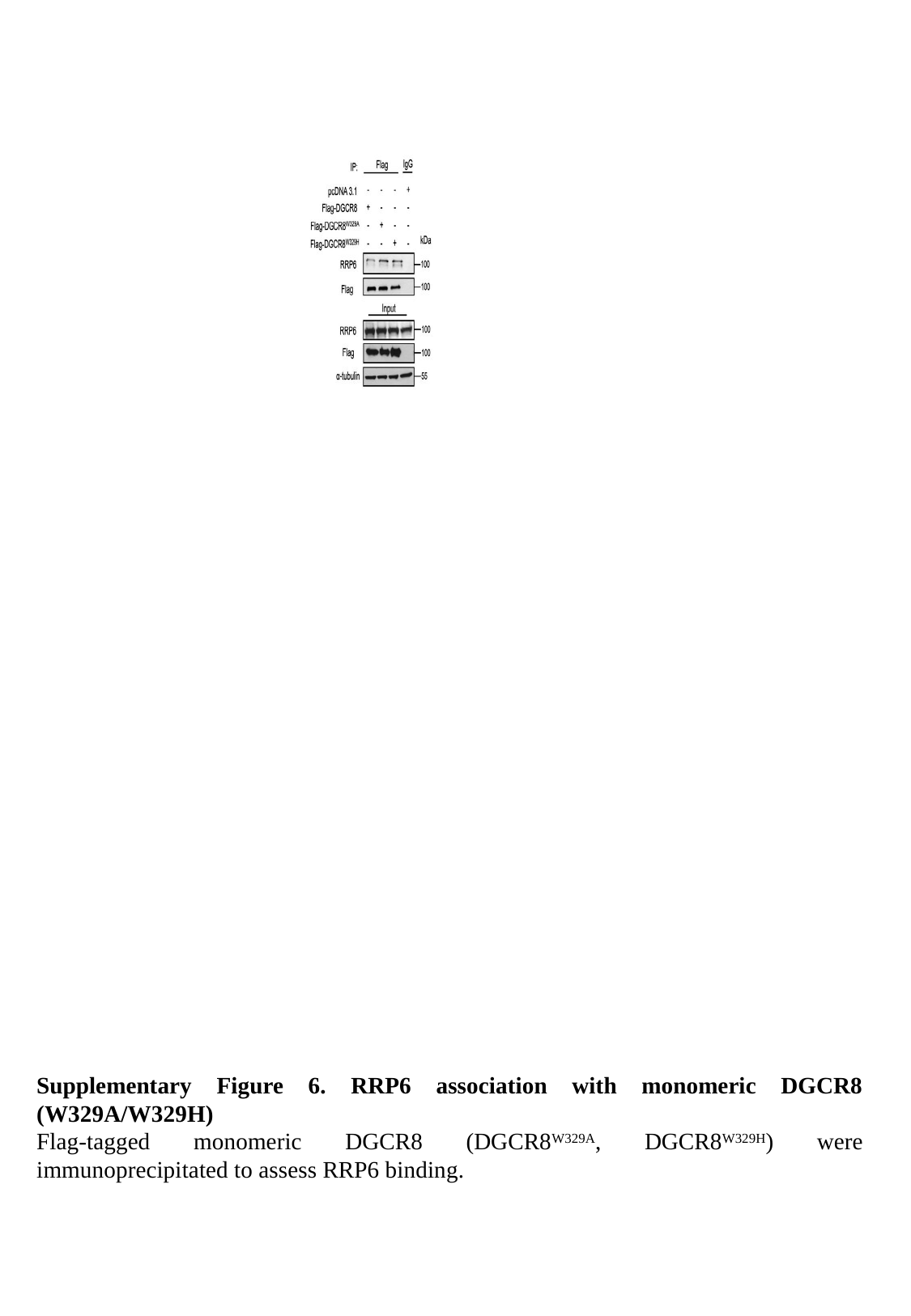

Supplementary Figure 6. RRP6 association with monomeric DGCR8 (W329A/W329H)
Flag-tagged monomeric DGCR8 (DGCR8W329A, DGCR8W329H) were immunoprecipitated to assess RRP6 binding.

## Slide 7
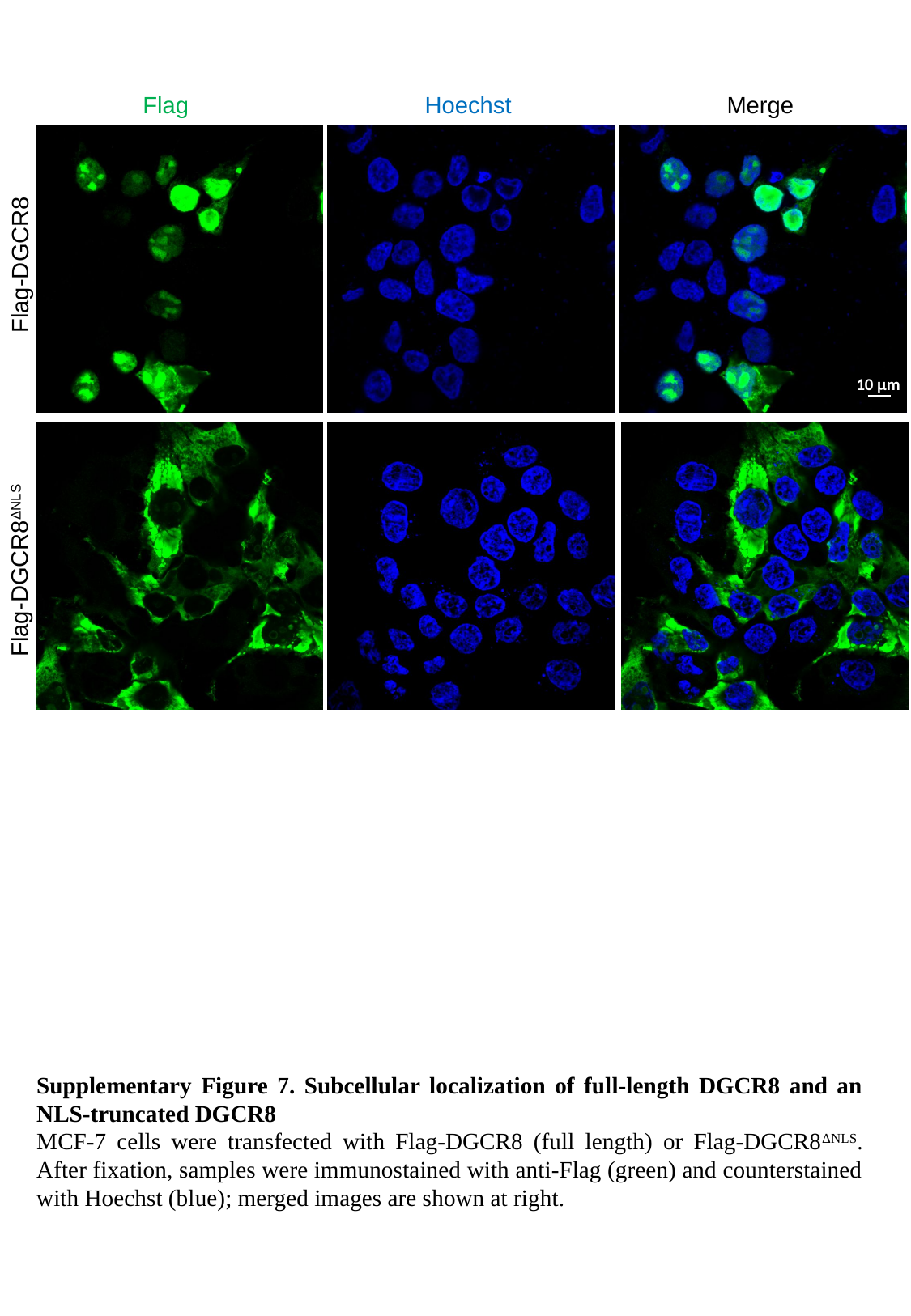

Flag
Hoechst
Merge
Flag-DGCR8
10 µm
Flag-DGCR8ΔNLS
Supplementary Figure 7. Subcellular localization of full-length DGCR8 and an NLS-truncated DGCR8
MCF-7 cells were transfected with Flag-DGCR8 (full length) or Flag-DGCR8ΔNLS. After fixation, samples were immunostained with anti-Flag (green) and counterstained with Hoechst (blue); merged images are shown at right.

## Slide 8
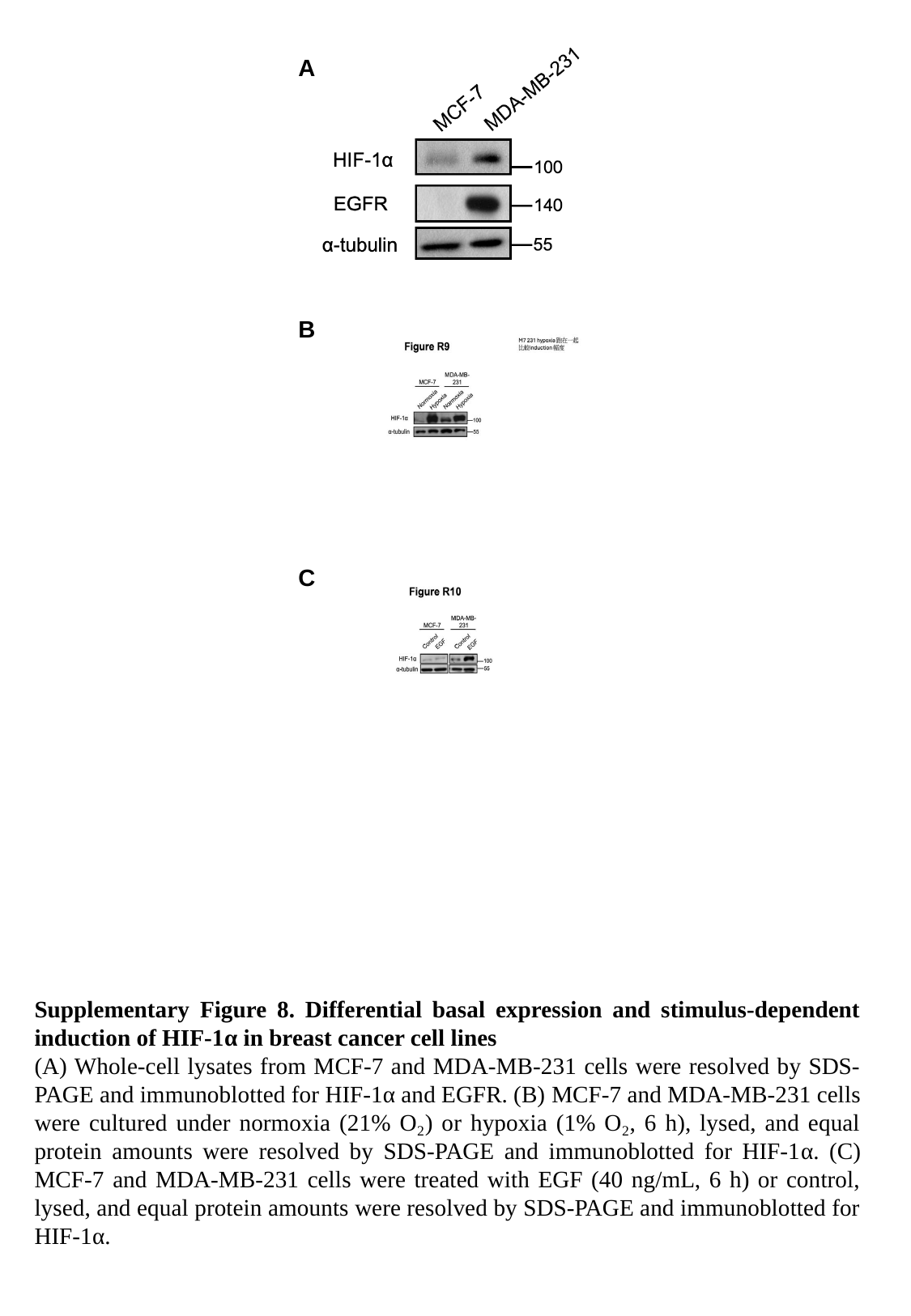

A
B
C
Supplementary Figure 8. Differential basal expression and stimulus-dependent induction of HIF-1α in breast cancer cell lines
(A) Whole-cell lysates from MCF-7 and MDA-MB-231 cells were resolved by SDS-PAGE and immunoblotted for HIF-1α and EGFR. (B) MCF-7 and MDA-MB-231 cells were cultured under normoxia (21% O₂) or hypoxia (1% O₂, 6 h), lysed, and equal protein amounts were resolved by SDS-PAGE and immunoblotted for HIF-1α. (C) MCF-7 and MDA-MB-231 cells were treated with EGF (40 ng/mL, 6 h) or control, lysed, and equal protein amounts were resolved by SDS-PAGE and immunoblotted for HIF-1α.

## Slide 9
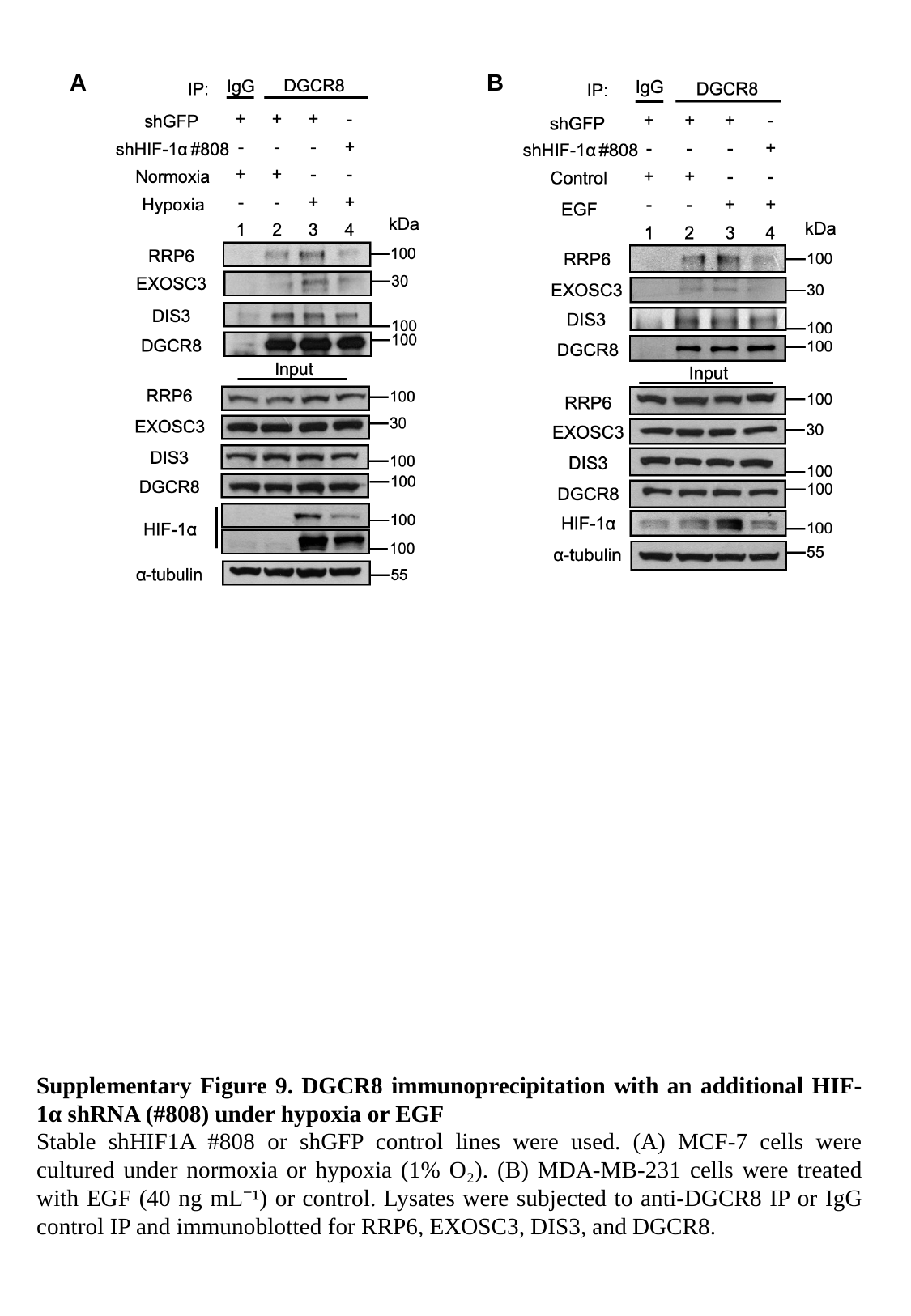

A
B
Supplementary Figure 9. DGCR8 immunoprecipitation with an additional HIF-1α shRNA (#808) under hypoxia or EGF
Stable shHIF1A #808 or shGFP control lines were used. (A) MCF-7 cells were cultured under normoxia or hypoxia (1% O₂). (B) MDA-MB-231 cells were treated with EGF (40 ng mL⁻¹) or control. Lysates were subjected to anti-DGCR8 IP or IgG control IP and immunoblotted for RRP6, EXOSC3, DIS3, and DGCR8.

## Slide 10
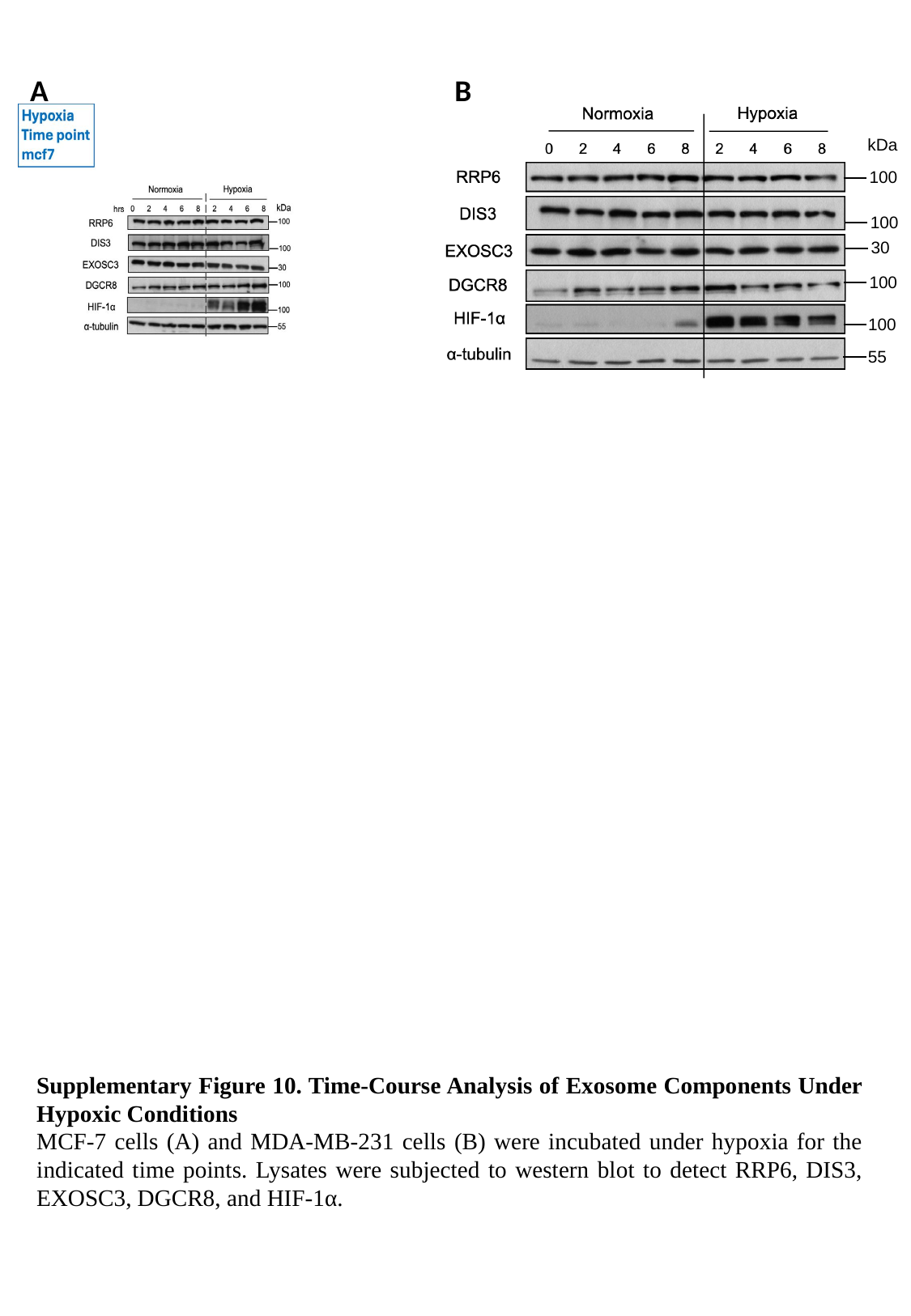

A
B
kDa
100
100
30
100
100
55
Supplementary Figure 10. Time-Course Analysis of Exosome Components Under Hypoxic Conditions
MCF-7 cells (A) and MDA-MB-231 cells (B) were incubated under hypoxia for the indicated time points. Lysates were subjected to western blot to detect RRP6, DIS3, EXOSC3, DGCR8, and HIF-1α.

## Slide 11
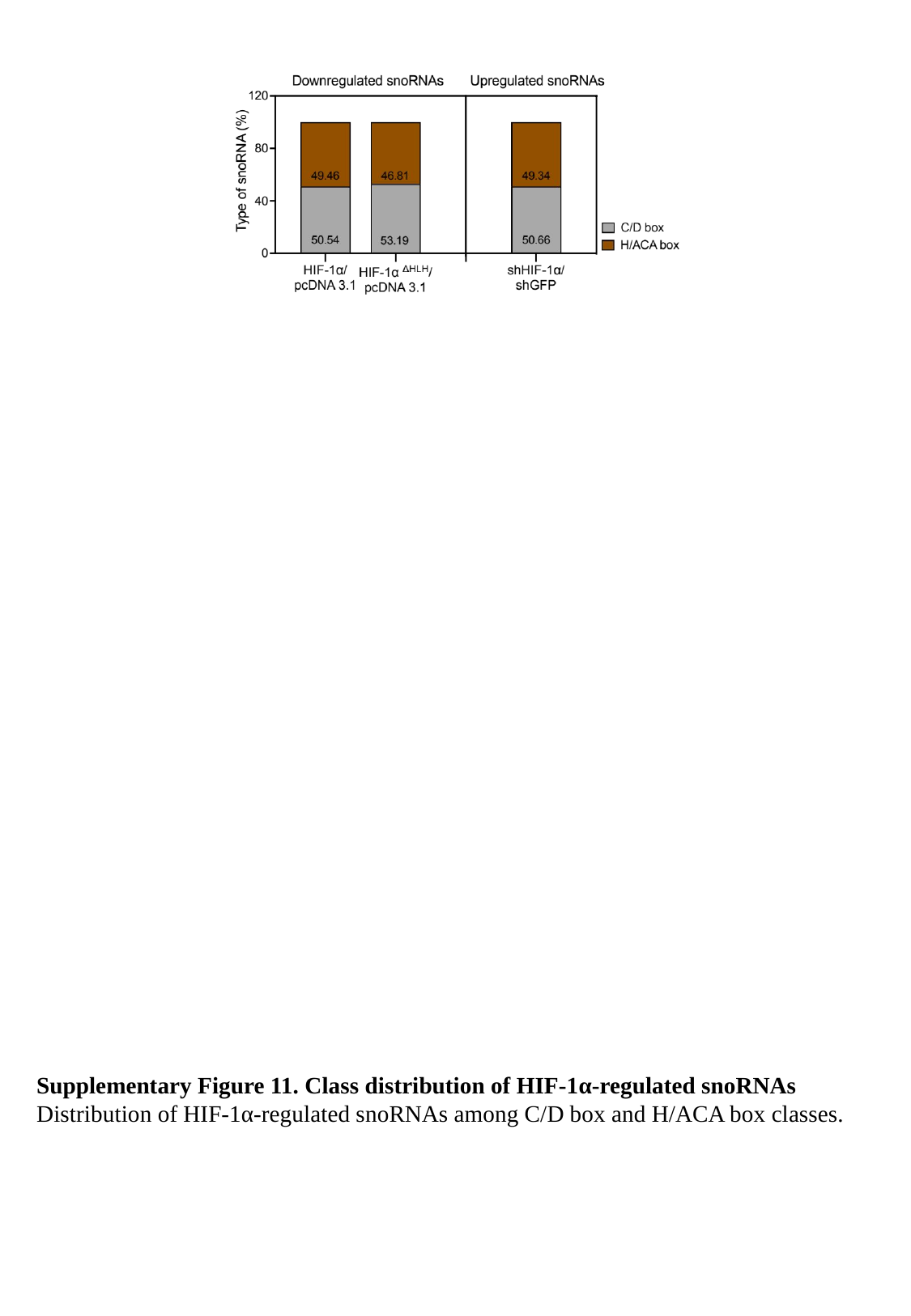

Supplementary Figure 11. Class distribution of HIF-1α-regulated snoRNAs
Distribution of HIF-1α-regulated snoRNAs among C/D box and H/ACA box classes.

## Slide 12
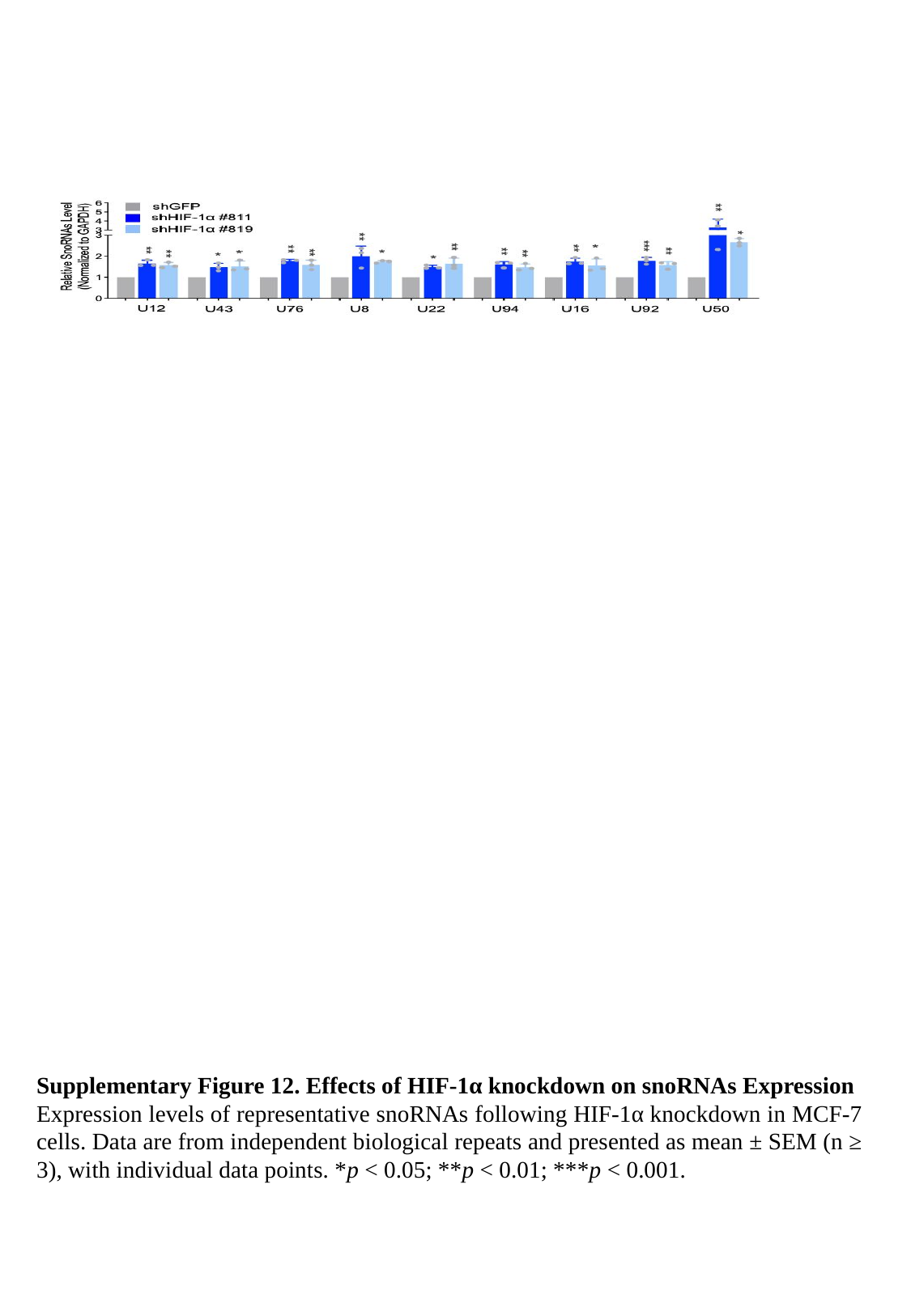

Supplementary Figure 12. Effects of HIF-1α knockdown on snoRNAs Expression
Expression levels of representative snoRNAs following HIF-1α knockdown in MCF-7 cells. Data are from independent biological repeats and presented as mean ± SEM (n ≥ 3), with individual data points. *p < 0.05; **p < 0.01; ***p < 0.001.

## Slide 13
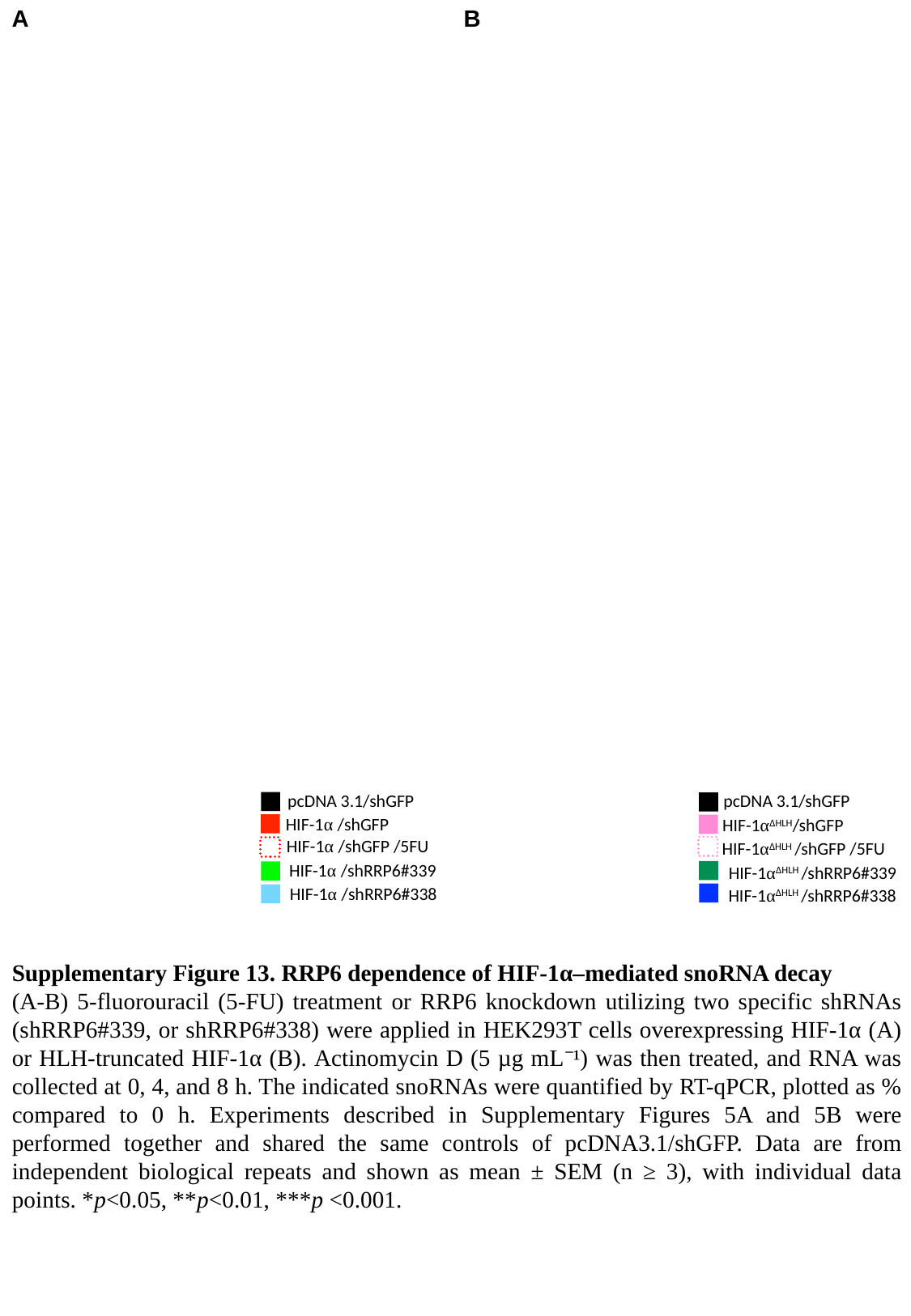

A
pcDNA 3.1/shGFP
HIF-1α /shGFP
HIF-1α /shGFP /5FU
HIF-1α /shRRP6#339
HIF-1α /shRRP6#338
B
pcDNA 3.1/shGFP
HIF-1αΔHLH/shGFP
HIF-1αΔHLH /shGFP /5FU
HIF-1αΔHLH /shRRP6#339
HIF-1αΔHLH /shRRP6#338
Supplementary Figure 13. RRP6 dependence of HIF-1α–mediated snoRNA decay
(A-B) 5-fluorouracil (5-FU) treatment or RRP6 knockdown utilizing two specific shRNAs (shRRP6#339, or shRRP6#338) were applied in HEK293T cells overexpressing HIF-1α (A) or HLH-truncated HIF-1α (B). Actinomycin D (5 µg mL⁻¹) was then treated, and RNA was collected at 0, 4, and 8 h. The indicated snoRNAs were quantified by RT-qPCR, plotted as % compared to 0 h. Experiments described in Supplementary Figures 5A and 5B were performed together and shared the same controls of pcDNA3.1/shGFP. Data are from independent biological repeats and shown as mean ± SEM (n ≥ 3), with individual data points. *p<0.05, **p<0.01, ***p <0.001.
