# Supplementary material for: A non-canonical role for HIF-1α: redirecting DGCR8 to the RNA exosome for snoRNA degradation and translational modulation
Source: Nucleic Acids Res. 2026 Feb 2;54(3):gkag070. doi: 10.1093/nar/gkag070 (PMC12862392; doi:10.1093/nar/gkag070)
Supplement: gkag070_Supplemental_Files [file gkag070_supplemental_files.zip › Supplementary Table 1-2_20250714_clean.docx]

**Supplementary Table 1. Primer information**

| Target | Primer Sequences |
| --- | --- |
| GAPDH | Forward:  GTCAGTGGTGGACCTGACCT  Reverse:  AGGGGTCTACATGGCAACTG |
| Pri-miR-125b | Forward:  ACCTCGAACAGAAATTGCCT  Reverse:  CACAAGTTAGGGTCTCAGGGA |
| Pri-miR-205 | Forward:  CCTTTGGCCAGGTAATGCAG  Reverse:  GCCTCCTGAACTTCACTCCA |
| Pri-let-7d | Forward:  TGCCAAGTAGAAGACCAGCA  Reverse:  AGAAAGGCAGCAGGTCGTAT |
| U16 | Forward:  TGCCTGCTGTCAGTAAGCTG  Reverse:  TGCTCAGTAAGAATTTTCGTCAA |
| U92 | Forward:  GTCACCATGCCTCCCTAGAA  Reverse:  ATCTGTCTGCCCCGTATCTG |
| U12 | Forward:  TTT CCC CAT CAG ATC GAC CC  Reverse:  AGA CAA AAC TGG CCA ATA GTG T |
| U43 | Forward:  GAA CTT ATT GAC GGG CGG AC  Reverse:  TCA GAA CGT GAC AAT CAG CAC |
| U76 | Forward:  TGC TAC TCT TGA GTG CTA GAA TG  Reverse:  GCC TCA GTT AAG ATA ATG GTG GT |
| U8 | Forward:  CTT ACC TGT TCC TCC TCC GG  Reverse:  TCA GAC AGG AGC AAT CAG GG |
| U22 | Forward:  GTC CCA GAG CCT GTA AAG GT  Reverse:  TCC CTC AGA CAG TTC CTT CT |
| U94 | Forward:  GCT GAG TCA TGG GAG CTG AA  Reverse:  CCA CAT GCA GGA CAA AGG AG |
| U50 | Forward:  TAT CTG TGA TGA TCT TAT CCC GAA CCT GAA C  Reverse:  ATC TCA GAA GCC AGA TCC GTA A |
| Pre-U16 | Forward:  GAT GGA ATT TAG CGG CCT GG  Reverse:  CGT CAA CCT TCT GTA CCA GC |
| Pre-U92 | Forward:  CAG ATA CGG GGC AGA CAG AT  Reverse:  ATCTGTCTGCCCCGTATCTG |
| Pre-U12 | Forward:  GAC TGC GAC AAG AAA GAG GC  Reverse:  AGA AAG CCC AGT TCC CAT GA |
| Pre-U43 | Forward:  GAA CTT ATT GAC GGG CGG AC  Reverse:  CAC ACA GGC CAA AGA ACT CC |
| Pre-U76 | Forward:  TGT GCC ACA ATG ATG ACA GT  Reverse:  CCA TTT TGG GTG CCT CAG TT |
| Pre-U8 | Forward:  GCT CAT ACC AGC GCG TTA TG  Reverse:  GTT CTA ATC TGC CCT CCG GA |
| Pre-U22 | Forward:  TGT TCA CTC CAG GCT GAA GT  Reverse:  CT GGG ACT AGG ACA GAG AGT |
| Pre-U94 | Forward:  GGC TCA CAC GTG TCT CTC TA  Reverse:  CCA CAT GCA GGA CAA AGG AG |
| Pre-U50 | Forward:  TCT GGC TTC TGA GAT GGA CC  Reverse:  GGT CCA TCT CAG AAG CCA GA |
| ZFAS1 | Forward:  CCA TTC GTT CTT TCG CGT CT  Reverse:  ACC AGA GGT CTC CAA CGA AG |
| RPL3 | Forward:  TAC AAG GCT GGC ATG ACT CA  Reverse:  ACA GTC TTG AAG GTC CGG AG |
| GAS5 | Forward:  GTC ACG CAT GCA CCA TAT GT  Reverse:  CAG GGC CTA CAA GTT GAG GA |
| TMTM107 | Forward:  TTT TGT CTT CTG CAG TGC CC  Reverse:  TTT TCA GCC CAA AGA CGG TG |
| SNHG1 | Forward:  GTG CTT AAA ACG GTG CTC CA  Reverse:  GGT TCC CTA TGT CCT GCC TT |
| PTCD3 | Forward:  AAG CCA ACC CAT CAG ACA GA  Reverse:  GGG AAG GGC TGT TAG ACA CT |
| SNHG5 | Forward:  AAA ACG CCT TGG AGT GTG AC  Reverse:  TGA AGA CAG CGC CAT TGT TC |
| RPL4 | Forward:  ATA GAT GTG TCG TGG AGG CC  Reverse:  TTC ACT CTA CGA TGC CAA CG |
| FAM29A | Forward:  AGC GAA ACT CCG TCT CAA AA  Reverse:  CTC CAC AAC TTG AAC ACA GGT |
| SNX12 | Forward:  GAATGAACGCTGCCTACACA  Reverse:  TTCCTGTCAATTGCCTCCTC |
| DLG5 | Forward:  GGCCAAGAAATCCTGTGATG  Reverse:  GAGTAAGGTGCCCACTCCTG |
| MALAT1 | Forward:  GACGGAGGTTGAGATGAAGC  Reverse:  ATTCGGGGCTCTGTAGTCCT |
| RTL-P primers | |
| 5.8s rRNA  Primer 1 | P1_Fu: GTGCGTCGATGAAGAACGC  P1_Fd: ATCGACACTTCGAACGCACT  P1_Rud: GACGCTCAGACAGGCGTAG |
| 18s rRNA  Primer 1 | P1_Fu: TACCTGGTTGATCCTGCCAGT  P1_Fd: CATTCGAACGTCTGCCCTAT  P1_Rud: GTTTCTCAGGCTCCCTCTCC |
| 18s rRNA  Primer 2 | P2_Fu: GCCTACCATGGTGACCACGGG  P2_Fd: ACCGCAGCTAGGAATAATGG  P2_Rud: CCCTCTTAATCATGGCCTCA |
| 18s rRNA  Primer 3 | P3_Fu: TTGGTTTTCGGAACTGAGGC  P3_Fd: TCAGATACCGTCGTAGTTCCG  P3_Ru: GCTTTGCAACCATACTCCCC  P3_Rd: GCTTTGCAACCATACTCCCC |
| 18s rRNA  Primer 4 | P4_Fu: AAAGTCTTTGGGTTCCGGGG  P4_Fd: AAAGGAATTGACGGAAGGGC  P4_Ru: CAGACAAATCGCTCCACCAA  P4_Rd: GGTTTCCCGTGTTGAGTCAA |
| 18s rRNA  Primer 5 | P5_Fu: TAACGAACGAGACTCTGGCA  P5_Fd: CGCTACACTGACTGGCTCA  P5_Ru: GGAATTCCTCGTTCATGGGG  P5_Rd: AATGGGGTTCAACGGGTTAC |
| 18s rRNA  Primer 6 | P6_Fu: GGGTCATAAGCTTGCGTTGA  P6_Fd: AAGTCCCTGCCCTTTGTACA  P6_Ru: CGAGGGCCTCACTAAACCA  P6_Rd: AGGGCCTCACTAAACCATCC |
| 18s rRNA  Primer 7 | P7_Fu: GGATGGTTTAGTGAGGCCCT  P7_Fd: GGATGGTTTAGTGAGGCCCT  P7_Ru: GTTCACCTACGGAAACCTTGT  P7_Rd: AGTTCGACCGTCTTCTCAGC |
| 28s rRNA  Primer 1 | P1_Fud: AAGTCCTTCTGATCGAGGCC  P1_Rd: GGAGTTTACCACCCGCTTTG  P1_Ru: ACGCCCTCTTGAACTCTCTC |
| 28s rRNA  Primer 2 | P2_Fu: AGGTTCTCTCGGGGCCAC  P2_Fd: CAAGGAGTCTAACACGTGCG  P2_Rud: TTCACCTTCATTGCGCCAC |
| 28s rRNA  Primer 3 | P3_Fu: CAAGGAGTCTAACACGTGCG  P3_Fd: CAAGGAGTCTAACACGTGCG  P3_Ru: GAGGGAAACTTCGGAGGGAA  P3_Rd: TTCACCTTCATTGCGCCAC |
| 28s rRNA  Primer 4 | P4_Fu: CTAGTAGCTGGTTCCCTCCG  P4_Fd: CGCTCATCAGACCCCAGAAA  P4_Ru: CCACCGTCCTGCTGTCTATA  P4_Rd: ACCGTCCTGCTGTCTATATCA |
| 28s rRNA  Primer 5 | P5_Fu: GTGTGGTGTGCGTCGGAG  P5_Ru: CGGCCTTCAAAGTTCTCGTT  P5_Rd: CTACTCGTCGCGGCGTAG |
| 28s rRNA  Primer 6 | P6_Fu: AACGAGAACTTTGAAGGCCG  P6_Fd: GTTGAACATGGGTCAGTCGG  P6_Rud: TCTGAACCCGACTCCCTTTC |
| 28s rRNA  Primer 7 | P7_Fu: CTCTCGCTGGCCCTTGAAA  P7_Fd: GTAAGGGAAGTCGGCAAGC  P7_Rud: AGAGCCAATCCTTATCCCGA |
| 28s rRNA  Primer 8 | P8_Fu: CGCCTAGCAGCCGACTTA  P8_Fd: TGCTCTGAATGTCAAAGTGAAGA  P8_Ru: TCATAGTTACTCCCGCCGTTT  P8_Rd: AGTTACTCCCGCCGTTTACC |
| 28s rRNA  Primer 9 | P9_Fu: GCCAAATGCCTCGTCATCTA  P9_Fd: GGAATCAGCGGGGAAAGAAG  P9_Rud: TTCACCGTGCCAGACTAGAG |
| 28s rRNA  Primer 10 | P10_Fu: GCACGGTGAAGAGACATGAG  P10_Fd: GTGTAGAATAAGTGGGAGGCC  P10_Ru: TTTCTGTCCTCCCTGAGCTC  P10_Rd: GTATTTCACCGGCGGCCC |
| 28s rRNA  Primer 11 | P11_Fu: CCTCACGATCCTTCTGACCT  P11_Fd: TGTATGTGCTTGGCTGAGGA  P11_Ru: TGCTACGTACGAAACCCCG  P11_Rd: GGGCGGGATTCTGACTTAGA |

**Supplementary Table 2. Antibodies information**

| **Target protein** | **Antibody** | **Applications** |
| --- | --- | --- |
| Drosha | *#3364(Cell signaling)* | WB, |
|  | *Sc-33778 (Santa cruz)* | PLA |
| DGCR8 | *A302-469A(Bethyl)* | WB, IP, PLA |
|  | *H00054487-B01P(Abnova)* | PLA, IF |
| HIF-1α | *Ab2185(Abcam)* | WB |
|  | *NB100-105 (Novus)* | WB, IP |
| RRP6 | *GTX107856(GeneTex)* | WB, PLA, IF |
| EXOSC3 | *GTX116123(GeneTex)* | WB |
| DIS3 | *GTX115645(GeneTex)* | WB |
| FLAG tag | *A2220 (Sigma)* | WB, IP |
| FBL | *GTX117997 (GeneTex)* | WB |
| Pseudouridine | *Code No.D347-3(MBL)* | RNA dot blot |
| Puromycin | *3RH11(Kerafast)* | WB |
| α-tubulin | *T9026 (Sigma)* | WB |
